# Fatty Acids and Volatile Flavor Components of Adipose Tissue from Local Tibetan Sheep in Qinghai with Dietary Supplementation of Palm Kernel Meal (PKM)

**DOI:** 10.3390/ani14142113

**Published:** 2024-07-20

**Authors:** Ying Ma, Lijuan Han, Shengzhen Hou, Linsheng Gui, Shengnan Sun, Zhenzhen Yuan, Chao Yang, Zhiyou Wang, Baochun Yang

**Affiliations:** College of Agriculture and Animal Husbandry, Qinghai University, Xining 810016, China; 13734619141@163.com (Y.M.); 1987990009@qhu.edu.cn (S.H.); 2017990039@qhu.edu.cn (L.G.); 2016980007@qhu.edu.cn (S.S.); 2017990038@qhu.edu.cn (Z.Y.); yangchao@qhu.edu.cn (C.Y.); 1992990011@qhu.edu.cn (Z.W.); 19083201126@qhu.edu.cn (B.Y.)

**Keywords:** tibetan sheep, PKM, flavor profile, subcutaneous fat, tail fat, intermuscular fat

## Abstract

**Simple Summary:**

Simple Summary: Currently, there is an urgent need to develop novel protein feeds to replace traditional soybean meal from the point of view of feed source, nutrition and cost. Palm kernel meal (PKM) is a potential alternative to traditional soybean meal. The present study evaluated the effect of PKM supplementation in traditional diets of Tibetan sheep on the flavor and fatty acid profile of different parts of adipose tissue of Tibetan sheep. The results showed that the addition of PKM resulted in the deposition of favorable volatile flavor compounds in Tibetan sheep fat, which effectively improved the fat flavor of Tibetan sheep. This finding provides a basis for the improvement of fat flavor in Tibetan sheep and the rational development of PKM feed.

**Abstract:**

Substituting traditional protein feed with palm kernel meal (PKM) in the diet of Tibetan sheep can be a cost-effective feeding strategy. To determine the impact of PKM on flavor development in different adipose tissues of Tibetan sheep, subjects were fed with 15% and 18% of PKM, while the control group received no PKM. The fatty acids and volatile compounds in the samples were then analyzed by GC-MS and HS-GC-IMS. Adding PKM to the diet significantly increased the C12:0, C14:0, C16:0 and C18:1N9 content in adipose tissues compared with the control, and most of these were associated with flavor formation (*p* < 0.05). The flavor compounds in the adipose tissues predominantly consisted of alcohols, ketones, acids and aldehydes. In particular, including PKM in the diet increased the proportion of ketones but decreased the proportion of alcohols, acids and aldehydes in subcutaneous and tail fat. Specifically, the proportion of acetone, acetoin monomer, 2,3-butanedione, 2-butanone monomer, 2-methyl-2-propanol, 2-methyl-2-propanol and methyl acetate increased significantly in the subcutaneous and tail fat (*p* < 0.05), while that of ethanol, 1-propanol monomer, butanol monomer, acetic acid monomer and acetic acid monomer decreased. Intermuscular fat exhibited variable results, mainly because the addition of PKM resulted in higher proportions of alcohols, including ethanol, 1-propanol and butanol monomer, especially at 15% PKM. In summary, the addition of PKM improved the flavor of Tibetan sheep fat and increased the amount of favorable volatile flavor compounds. This study can serve as reference for understanding the effects of dietary PKM on the adipose tissue flavor profile of Tibetan sheep.

## 1. Introduction

In the unique geographic, human environment and resource conditions of Qinghai, Tibetan sheep (*Ovis aries* L.) is regarded as a distinctive local brand known for its rich nutritional profile, delicate and tender meat, minimal gaminess and highly aromatic mutton that boasts a subtle grass flavor. This reputation for excellence in the Northwest has, in fact, been acknowledged in different studies [1,2]. The exceptional flavor of Qinghai Tibetan sheep meat not only provides sensory pleasure, but also enhances the digestion and absorption of nutrients, which is an important factor in evaluating lamb quality. Previous research suggested that the characteristic flavor of lamb meat was primarily associated with adipose tissue [3,4]. In fact, historical practices in Asia and some Middle Eastern countries involved using specific parts of fatty tissue for preparing dishes and meat products, as it was believed that this animal fat contributed a species-specific flavor that enhanced the palatability of the food [5].

Flavor is manifested through volatile aroma and non-volatile taste compounds, with meat composition, especially the fat content, influencing the release of flavor during cooking and serving [6]. Adipose acts as a solvent for flavor compounds, and it interacts with other meat components to influence the release of flavor compounds [6]. In addition, through hydrolysis, oxidation, esterification with other compounds or the Meladic reaction, adipose tissues yield various flavor compounds [7], with the volatile fatty acids being particularly responsible for the characteristic flavor of lamb meat [8]. These reports suggest that the content and composition of fatty acids (FA) and volatile compounds (VC) are the two most important parameters for assessing the quality of adipose tissue and meat. However, there are significant variations in the fatty acid composition and flavor components of adipose tissues from different parts of the body. For example, Güler [9] and Peng et al. [10] found that the tail fat of sheep had the lowest SFA content but the highest amount of MUFA and PUFA. Furthermore, that tail fat was particularly rich in fatty acid oxidation products such as heptanal, octanal, and nonanal. Castro et al. mentioned that the percentage of stearic acid (C18:0) content in intramuscular and subcutaneous fat of growing lambs were close to each other, whereas subcutaneous fat stored more C18:1 [11]. In contrast, the dorsal subcutaneous fat of boars contained a higher proportion of SFA and UFA compared to their abdominal subcutaneous fat and perirenal fat [12].

Diet significantly influences the composition and content of animal adipose tissue, especially the fatty acids and volatile flavor compounds (VOCs) substances. For instance, it was reported that, in lambs, cardoon meal could reduce the “animal/barnyard odor” in their kidney fat [13], while alfalfa consumption could increase the concentration of perirenal fat skatole as opposed to those that consumed only cocksfoot [14]. In addition, grazing in highland pastures increased the C18:0 content in animal adipose tissue due to the abundance of grass species [15]. Similarly, PKM is considered to be an excellent plant-based protein feed, and its supplementation in the diet of Tibetan sheep can not only reduce feed costs, but also help to fulfill the objectives of the “soybean meal reduction and substitution program”, while improving Tibetan sheep meat quality [16,17]. In fact, the current authors previously demonstrated that PKM influenced muscle flavor, but studies are still lacking regarding its effects on the active compounds responsible for fat flavor in Tibetan sheep [18].

Based on the above background, this experiment used gas chromatography-mass spectrometry (GC-MS) and headspace gas ion mobility spectrometry (HS-GC-IMS) to analyze in depth the differences in fatty acids and VOCs in different parts of the fat of Tibetan sheep after feeding palm meal. This study lays the foundation for the flavor regulation of Tibetan sheep fat and meat, which can reveal the potential of PKM in improving the quality of Tibetan sheep PKM in improving the quality of Tibetan sheep fat. The addition of PKM can achieve the purpose of directional regulation of fat flavor in Tibetan sheep, and then understand the contribution of different parts of fat to meat flavor, so as to improve the overall quality of Tibetan sheep products, and increase the added value and market competitiveness of livestock products [19]. On the other hand, it helps to clarify the optimal amount of PKM added in Tibetan sheep. By analyzing the effects of different PKM additions on fatty acids and VOCs in adipose tissue of Tibetan sheep and optimizing the feed formulation, it can improve the feed utilization rate and reduce the feed cost, which provides a theoretical basis for the development of new plant protein feeds.

## 2. Materials and Methods

### 2.1. Ethical Statement

All animal experiments in the present study were in accordance with animal welfare provisions approved by the Animal Care Committee of the Qinghai University of China (QUA-2020-0709).

### 2.2. Feeding Trial and Sampling

The feeding trials were conducted at the Black Tibetan Sheep Breeding Center in Guinan County, Tibetan Autonomous Prefecture, Qinghai Province. A total of 15 similar Tibetan sheep of similar weight and between 2–3 months of age (initial weight: 18 ± 3 kg) were randomly divided into 3 groups. After the 14-day adaptation period, all Tibetan sheep were fed at the same frequency in the same environment with 90 days. This feeding trial was divided into two phases, with a basal ration consisting of oat hay, oat silage, and a concentrate supplement. The first phase was 15–45 d with a concentrate: oat silage: oat hay ratio of 60:20:20, and the second phase was 45–105 d with a concentrate: oat silage: oat hay ratio of 70:15:15. Three experimental concentrate diets were formulated containing graded levels of palm kernel meal (PKM, 0%, 15% and 18%), as shown in Table 1. Table 2 demonstrates the composition and content of fatty acids in dietary PKM. Afterwards, all animals were slaughtered on the same day in the same commercial slaughterhouse by mechanical shock and bloodletting according to the European Union welfare guidelines. The slaughterhouse facilities met the requirements of the Institute of Animal Care and Use Committee. After slaughter, subcutaneous fat, tail fat and intermuscular fat were taken separately and immediately transferred to the laboratory for storage at −80 °C.

### 2.3. Fatty Acid Analysis

Modified by the method of Zhang et al. [2]: The lipids from the three adipose samples were extracted using a chloroform: methanol (2:1, *v*/*v*) mixture. Prior to extraction, the lipids underwent methyl esterification with a sulfuric acid-methanol solution and were then extracted with n-hexane. To serve as an internal standard, Methylnonadecanoat was added, mixed, and injected into the GC-MS detection vials. The separation of the samples occurred on an Agilent 19091S-433UI capillary column (Agilent Technologies, Inc., Santa Clara, CA, USA). The program began at 80 °C and the temperature was subsequently increased to 180 °C at a rate of 20 °C/min, followed by an increase to 280 °C at a rate of 5 °C/min. The carrier gas utilized in this study was helium, flowing at a rate of 1.0 mL/min. The mass spectrometry analysis was conducted using an Agilent 5977B MSD mass spectrometer. The transmission line, ion source, and inlet temperatures were set at 250 °C, 230 °C, and 280 °C, respectively. The analytes were detected employing an electron bombardment ionization (EI) source, with electron energy of 70 eV, operating in SCAN/SIM mode. The MSD ChemStation software (Agilent G1701EA) was employed to extract the peak area and retention time of the samples, construct a standard curve, and determine the FA content of the samples.

### 2.4. HS-GC–IMS Analysis

Referenced to the methodology of Ma et al. [20], GC-IMS technique combined with headspace injection for the determination of VOCs substances in different parts of adipose tissue. Putted 3 g of adipose tissue sample into a 20 mL headspace vial and incubate at 50 °C for 20 min. At the end of the incubation, 500 μL of constant headspace gas was injected into the GC-MS coupler and then separated on an RTX-5 (30 m × 0.53 mm × 1 μm; RESTEK, Bellefonte, PA, USA) capillary column with N_2_ (99.999%), and the programmed flow rates were as follows: 2 mL/min for 10 min, 10 mL/min for 10 min, and 100 mL/min for 10 min. IMS uses purified N_2_ at a flow rate of 150 mL/min as the drift gas, and the temperature of the drift tube is set to 45 °C. IMS was performed at atmospheric pressure and GC-IMS data were analyzed using the Laboratory Analysis Viewer (LAV V0.77.2) software and GC-IMS library search.

### 2.5. Identification and Quantification of Volatile Compounds

MS fragmentation patterns were compared with those of the National Institute of Standards and Technology (NIST) Mass Spectral Library (Version 14.0) to identify volatile compounds, and the retention indices (RI) were determined by analyzing a range of n-alkanes (C7–C30) using identical chromatographic conditions for comparison purposes. In addition, literature and online databases (https://www.flavornet.org; http://www.thegoodscentscompany.com/) were reviewed for odor descriptions, accessed on 1 Februray 2024. The quantitative analysis of volatile compounds was performed using an internal standard calibration method. 2-Methyl-3-heptanone was used as an internal standard, and the amount of each compound was calculated by comparing its area to the internal standard.

### 2.6. Calculations and Statistical Analysis

To identify the significant differences among fat quality, fatty acids and VOCs substances data were statistically analyzed using normality (Shapiro–Wilk’s W) and homogeneity of variance (Levine) tests. SPSS 20.0 was used, normally distributed data were analyzed by multiple sample analysis (ANOVA), non-normally distributed data were analyzed by non-parametric tests (Kruskal–Wallis test, Mann–Whitne test), and the results were expressed as mean ± SEM. In the statistical model, *p* < 0.05 was considered a significant difference. In addition, fatty acids and VOCs substances were subjected to joint Pearson correlation analysis.

## 3. Results

### 3.1. FAs Profiles of Adipose Tissues

#### 3.1.1. Effects of Three PKM Levels on the Composition and Content of FAs in Subcutaneous Fat, Tail Fat and Intermuscular Fat of Tibetan Sheep

**Figure 1:**
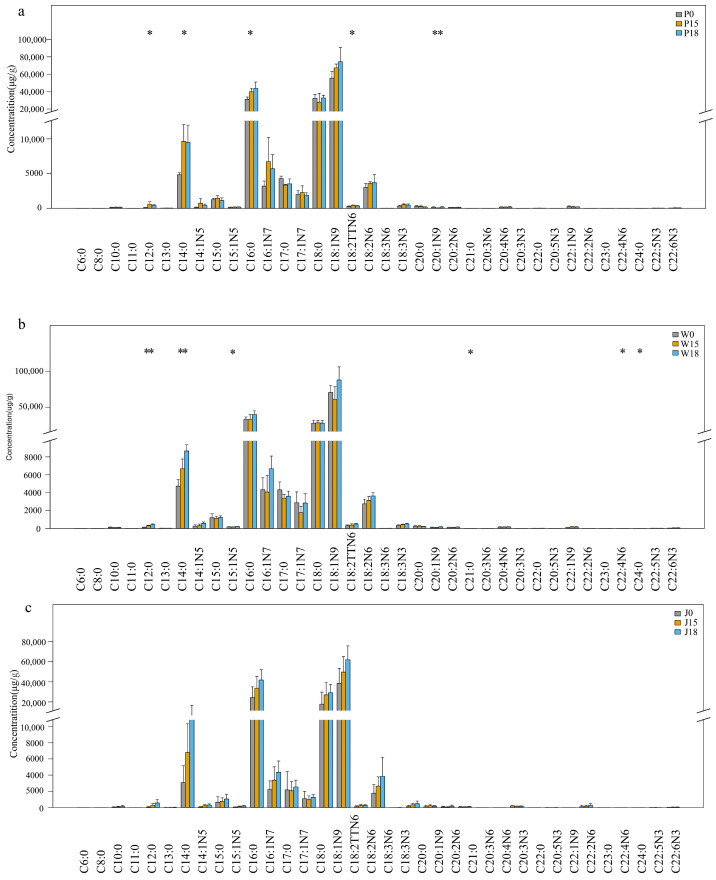
FAs Composition and Content Comparison Box Plot ((**a**) subcutaneous fat, (**b**) tail fat, (**c**) intermuscular fat). * denotes significant difference between groups (*p* < 0.05), ** denotes highly significant difference (*p* < 0.01).

Changes in the FAs composition and content of subcutaneous fat, tail fat and intermuscular fat, after supplementing Tibetan sheep diets with different levels of PKM, are shown in Figure 1, Table 3 and Tables S1–S3. Overall, C14:0, C16:0, C18:0 and C18:1N9 were the dominant fatty acids in all three adipose tissue sites. Specifically, in the subcutaneous fat, levels of C12:0, C14:0 and C16:0 were significantly higher in the PKM supplementation groups (P15, P18) compared with the blank group (P0), with the P18 group also exhibiting higher C16:0 levels than the P15 one (*p* < 0.05). In addition, feed supplementation with 15% PKM significantly increased the amount of C18:2TTN6 and reduced the C20:1N9 content (*p* < 0.05). In tail fat, the proportions of C12:0, C14:0 and C21:0 increased at higher levels of PKM, although the addition of 15% PKM also decreased the proportions of C24:0 and C15:1N5 (*p* < 0.05). Finally, in intermuscular fat, the amount of C12:0, C14:0, C16:0, C16:1N7, C18:0, C18:1N9 and C18:2N6 increased with increasing PKM levels in the feed, with the highest content reached in J18 (*p* > 0.05).

The supplementation of PKM in the diet also increased the levels of SFA, MUFA and PUFA in subcutaneous and intermuscular fat, with higher values observed in the P18 and J18 groups compared with the P15 and J15 ones (*p* > 0.05). However, in tail fat, even though the SFA and PUFA content still increased with increasing levels of dietary PKM, that of MUFA was actually reduced in W15 compared with the blank group (*p* > 0.05). In addition, significant differences in the N6/N3 ratios were observed in subcutaneous and tail fat, with the P15 group exhibiting a significantly lower ratio than the P0 group (*p* < 0.05). Similarly, both W15 and W18 had significantly lower N6/N3 ratios than W0 (*p* < 0.05).

#### 3.1.2. Differences in the Composition and Content of FAs in Different Parts of Adipose Tissue at the Same Level of PKM in Feeds

**Figure 2:**
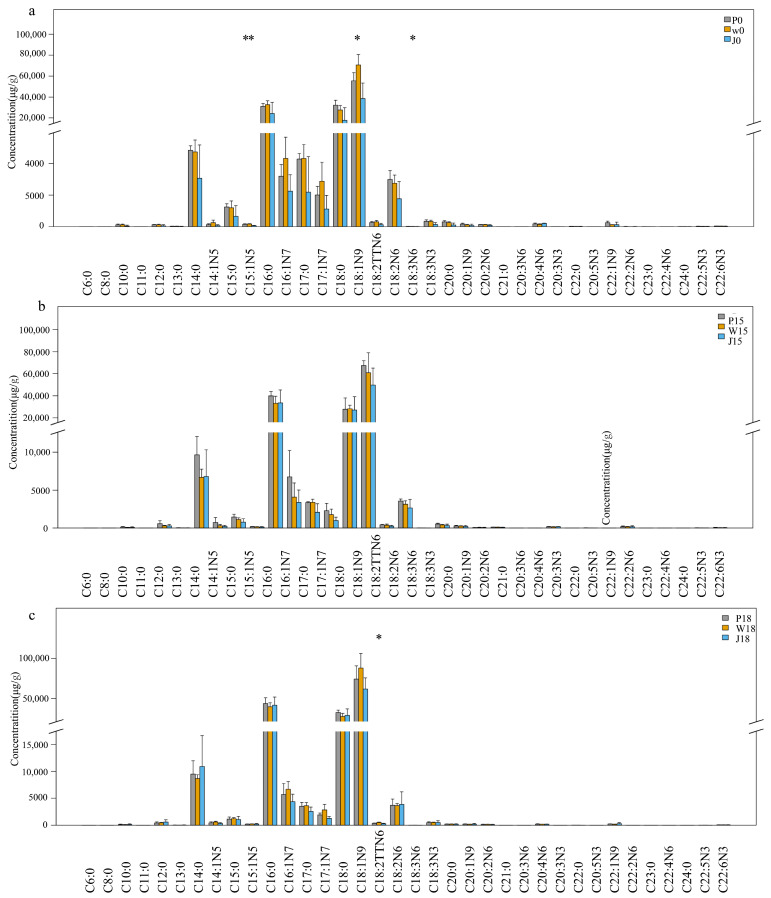
FAs Composition and Content Comparison Box Plot ((**a**) 0% PKM, (**b**) 15% PKM, (**c**)18% PKM). * denotes significant difference between groups (*p* < 0.05),** denotes highly significant difference (*p* < 0.01).

Under similar PKM feed conditions, variations were still noted in the FAs composition and content between the different parts of adipose tissue of Tibetan sheep as shown in Figure 2, Table 4 and Tables S4–S6. For instance, the amount of C18:1N9 was highest in the W0 group and lowest in the J0 group when no PKM was added to the diet, with the C15:1N5 and C18:3N6 content also lower in the J0 group compared with P0 and W0 (*p* < 0.05). Similarly, J0 had the lowest amount of some SFAs, such as C14:0, C15:0, C16:0, C17:0 and C18:0. After adding 15% PKM, the levels of different FAs (C14:0, C16:0, C18:0, C16:1N7, C17:1N7, C18:1N9 and C18:2N6) were reduced in both W15 and J15 groups compared to the blank group, although this difference was not statistically significant (*p* > 0.05). In contrast, the addition of 18% PKM to the diet only changed the amount of C18:2TTN6 between the three types of adipose tissue (W18 > J18, *p* < 0.05). In fact, the C14:0 content was higher in J18 than in P18 and W18, and similarly, that of C16:1N7, C17:0, C17:1N7 and C18:1N9 was higher in W18 than in P18 and J18. However, these differences were not significant (*p* > 0.05).

The SFA content was highest in subcutaneous fat regardless of the amount of PKM added to the feed (*p* > 0.05). Similarly, the subcutaneous fat had the highest amount of PUFA when no PKM was added, although the content was not different from that obtained after adding 15% PKM (*p* > 0.05). On the other hand, MUFA was highest in tail fat both at 0% and 18% PKM (*p* > 0.05). However, the N6/N3 ratios did not vary significantly between the adipose tissue of the three sites.

### 3.2. VOCs Compounds Composition and Content of Adipose Tissues

#### 3.2.1. Effects of Three PKM Levels on the Composition and Content of FCs in Subcutaneous Fat, Tail Fat and Intermuscular Fat of Tibetan Sheep

**Figure 3:**
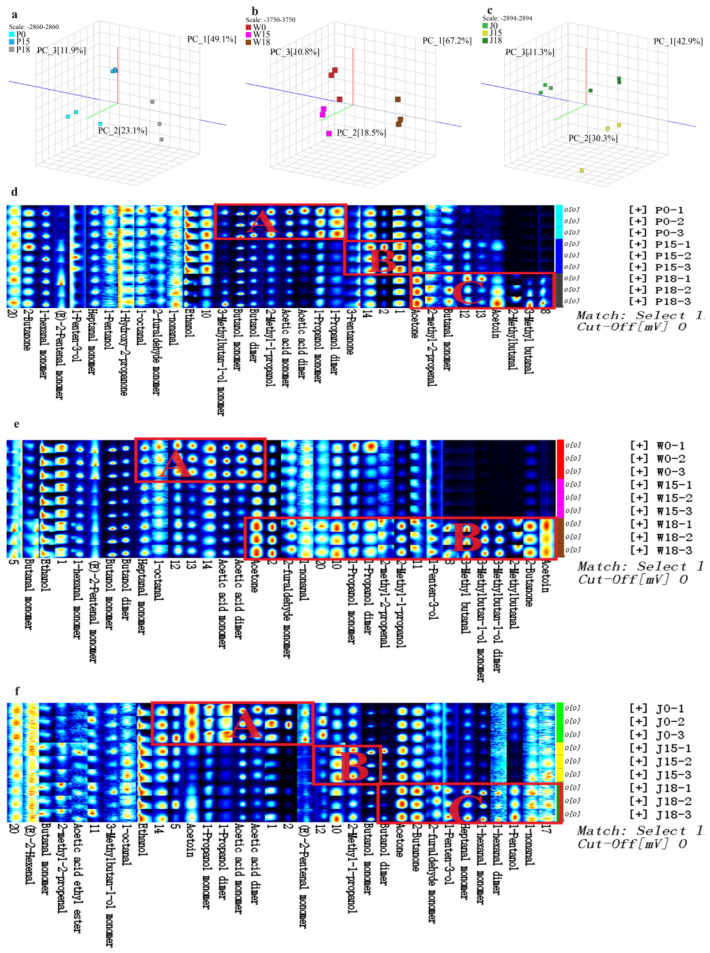
(**a**–**c**) graphs of PCA analysis of three adipose tissue samples, (**d**–**f**) fingerprints of VOCs substances; P: indicates subcutaneous fat, W: indicates tail fat, J: indicates intermuscular fat. Substances in the regions delineated by A, B and C in the figure have higher concentrations in their corresponding groups, e.g., substances in region A of the (**d**) figure have higher concentrations in the P0 group; each row of the graph represents all the signal peaks selected from one sample, and each column represents the signal peaks of the same VOCs in different samples; some substances are followed by -M and -D, which are the monomer and dimer of the same substance; the uncharacterizable substances are marked with arabic numerals (e.g., 1, 2, 3).

PCA plots of the samples, shown in Figure 3a–c, highlight significant separation of the three types of adipose tissue under varying PKM amounts in the feeds, thus suggesting the significant influence of different PKM levels on the VOCs compounds in Tibetan sheep. All the fingerprints presented in Figure 3d,e had 34 characteristic peaks that allowed differentiation between the types and concentrations of volatile sub-flavor substances in subcutaneous, tail and intermuscular fat. In particular, it was observed that, for the subcutaneous fat, the highest concentration of substances in regions A, B and C was found in P0, P15 and P18, respectively. Similarly, for tail fat, W0 and W18 had the highest concentration of substances in the regions A and B, respectively. Finally, in the case of intermuscular fat, substances in regions A, B and C were highest in concentration in samples J0, J15 and J18, respectively.

**Figure 4:**
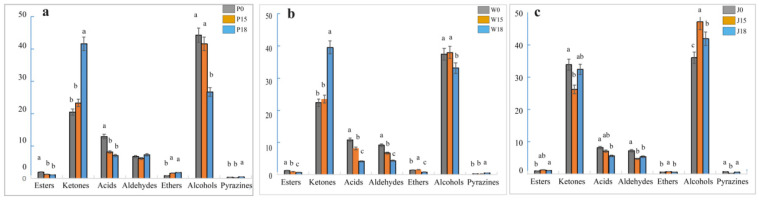
Percentage composition of VOCs in adipose tissue for each group. (**a**) subcutaneous fat; (**b**) tail fat; (**c**) intermuscular fat. lowercase letters: Same lowercase letters indicate insignificant differences (*p* > 0.05), different lowercase letters indicate significant differences (*p* < 0.05). P: indicates subcutaneous fat, W: indicates tail fat, J: indicates intermuscular fat, the different colors of the bar graph represent different parts of adipose tissue under different levels of PKM.

Further qualitative and quantitative analyses identified 39 VOCs substances, including 10 ketones, 7 aldehydes, 15 alcohols, 3 esters, 2 acids, 1 ether and 1 pyrazine, in the three types of adipose tissue (Figure 4 and Table 5). Specifically, in subcutaneous fat, the addition of 18% PKM significantly increased the percentage of ketones while decreasing the percentage of alcohols. Furthermore, the addition of PKM (15% and 18%) also decreased the percentage of esters and acids (*p* < 0.05). Some of the compounds for which the concentration increased significantly after adding 18% PKM were (E)-2-pentenal, 1-hydroxy-2-propanone, 2,3-butanedione, 2-butanone monomer, 2-methyl-2-propanol, 2-methylbutanal, 3-methyl butanal, acetoin dimer, acetoin monomer, acetone, dimethyl sulfide and methyl acetate. On the other hand, 1 -of propanol monomer, acetaldehyde, acetic acid monomer, ethanol and ethyl 2-hydroxypropanoate were some of those that exhibited a significant decrease in percentage (*p* < 0.05). Similarly, the addition of 15% PKM significantly increased the percentage of 1-penten-3-ol and 2-propanol dimer (*p* < 0.05). As far as tail fat was concerned, the addition of 18% PKM increased the percentage of ketones but decreased the percentage of alcohols. PKM also decreased the percentage of aldehydes, esters and acids at 15% and 18% (*p* < 0.05). In particular, adding 18% PKM significantly increased the amount of 1-hydroxy-2-propanone, 2,3-butanedione, 2,5-dimethylpyrazine, 2-butanone dimer, 2-methyl-1-propanol, 2-methyl-2- propanol, 2-methylbutanal, 2-pentanone, 3-methyl butanal, 3-methylbutan-1-ol monomer, acetoin dimer, acetoin monomer, acetone and methyl acetate, while reducing that of (E)-2-pentenal, 1-hexanal, 1-penten-3-ol, 1-propanol monomer, acetaldehyde, acetic acid dimer, acetic acid monomer, butanol monomer, ethanol, ethyl 2-hydroxypropanoate, heptanal and propanal (*p* < 0.05). Similarly, the addition of 15% PKM significantly increased the amount of 2-propanol monomer and dimethyl sulfide (*p* < 0.05). In the case of intermuscular fat, the addition of 15% and 18% PKM increased the proportion of alcohols but decreased the proportion of aldehydes and acids (*p* < 0.05). Specifically, the addition of 18% PKM significantly increased the percentage of 1-hexanal, 1-pentanol monomer, 1-penten-3-ol, 2-butanone dimer, 2-heptanone, acetone and heptanal, while decreasing the percentage of acetic acid monomer and acetic acid dimer (*p* < 0.05). Furthermore, the addition of 15% PKM increased the percentage of 3-methyl butanal, 2-methylbutanoic acid, methyl ester, 2-propanol dimer, butanol dimer, butanol monomer, ethanol and propanal (*p* < 0.05) but decreased that of (E)-2-pentenal, 2,3-butanedione, 1-propanol dimer, 1-propanol monomer, acetaldehyde, acetoin dimer, acetoin monomer, cyclopentanone and ethyl 2-hydroxypropanoate (*p* < 0.05).

#### 3.2.2. Differences in the Composition and Content of FCs in Different Parts of Adipose Tissue at the Same Level of PKM in Feeds

**Figure 5:**
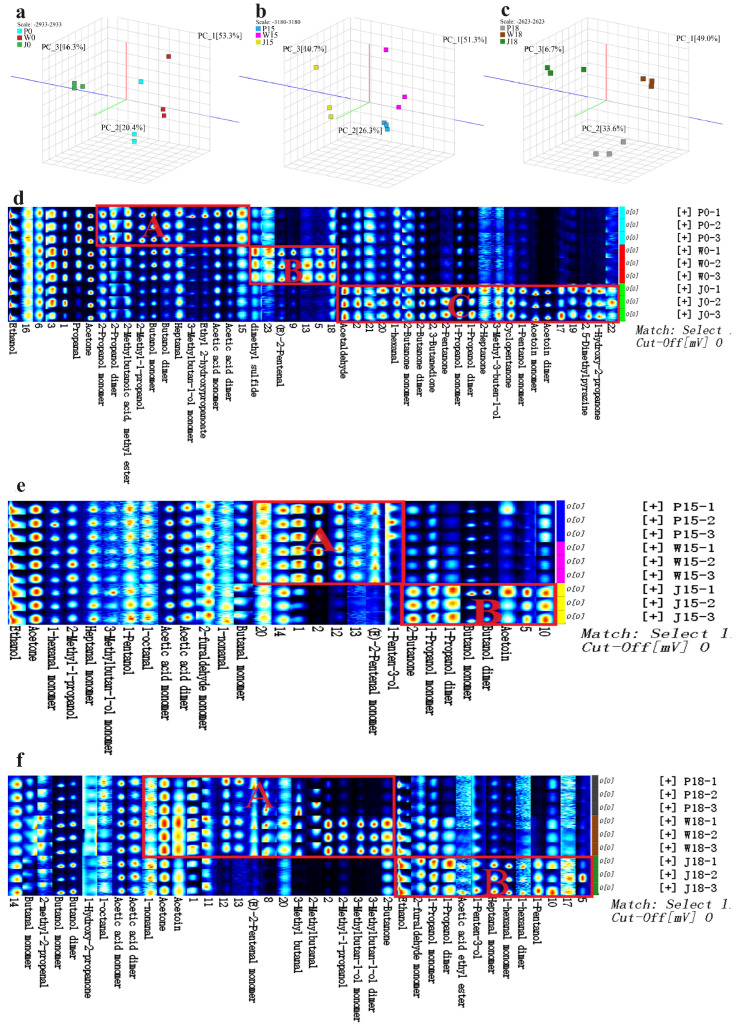
(**a**–**c**) graphs of PCA analysis of three adipose tissue samples, (**d**–**f**) fingerprints of VOCs substances; P: indicates subcutaneous fat, W: indicates tail fat, J: indicates intermuscular. Substances in the regions delineated by A, B and C in the figure have higher concentrations in their corresponding groups, e.g., substances in region A of the (**d**) figure have higher concentrations in the P0 group.

At similar PKM levels, the three types of adipose tissue in Tibetan sheep still differed in terms of their VOCs substances as shown in Figure 5a–c. For instance, when no PKM was added, the concentration of substances in regions A, B and C was higher in subcutaneous fat, tail fat and intermuscular fat, respectively. Furthermore, at 15% PKM levels, region A substances were enriched in both subcutaneous and tail fat, while those in region B were more concentrated in intermuscular fat. Finally, after adding 18% PKM, the concentration of region A substances was higher in tail fat, with intermuscular fat exhibiting a higher concentration of region B substances.

**Figure 6:**
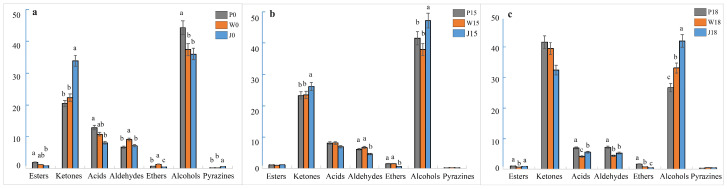
Percentage composition of VOCs in adipose tissue for each group. (**a**) 0%; (**b**) 15%; (**c**) 18%. lowercase letters: Same lowercase letters indicate insignificant differences (*p* > 0.05), different lowercase letters indicate significant differences (*p* < 0.05). P: indicates subcutaneous fat, W: indicates tail fat, J: indicates intermuscular fat, the different colors of the bar graph represent different parts of adipose tissue under the same level of PKM.

Differences in VOCs substances between the three types of adipose tissue are shown in Table 6 and Figure 6. When PKM was not added to the diet, intermuscular fat had a significantly higher proportion of ketones but a significantly lower amount of acids and alcohols compared with subcutaneous and tail fat. In addition, the proportion of aldehydes and ethers in tail fat was higher in comparison with the other two sites. In particular, cyclopentanone, acetoin monomer, acetoin dimer, 2-pentanone, 2-butanone dimer, 2,5-dimethylpyrazine, 2,3-butanedione, 1-propanol monomer, 1-propanol dimer, 1-pentanol monomer and 1-hydroxy-2-propanone were found in significantly higher proportions in intermuscular fat than in subcutaneous and tail fat (*p* < 0.05). Furthermore, the amount of propanal, methyl acetate, ethyl 2-hydroxypropanoate, ethanol, butanol monomer, butanol dimer, acetone, acetic acid monomer, 2-propanol monomer, 2-propanol dimer, 2-methylbutanoic acid, methyl ester, 2-methyl-2-propanol and 2-methyl-1-propanol ranged from high to low in subcutaneous fat, tail fat and intermuscular fat, respectively. At 15% PKM levels, intermuscular fat had significantly higher proportions of ketones and alcohols but significantly lower amounts of aldehydes and ethers than subcutaneous and tail fat (*p* < 0.05). Specifically, 1-propanol dimer, 1-propanol monomer, 2,3-butanedione, 2-butanone dimer, 2-methylbutanoic acid, methyl ester, butanol monomer and ethanol were present in decreasing amounts when comparing intermuscular fat, tail fat and subcutaneous fat, respectively (*p* < 0.05). At the same time, tail fat had higher proportions of (E)-2-pentenal, 2-methyl-1-propanol and acetaldehyde, while intermuscular fat had lower proportions of 1-hexanal, 1-penten-3-ol, 2-propanol dimer, 2-propanol monomer, dimethyl sulfide, heptanal, methyl acetate and propanal (*p* < 0.05). Supplementing the feeds with 18% PKM induced an opposite change to that of the blank group, with an increase in the proportion of alcohols in intermuscular fat (*p* < 0.05) as well as a decrease in the amount of acids, aldehydes and ethers in both tail fat and intermuscular fat (*p* < 0.05). In particular, intermuscular fat had significantly higher proportions of 1-hexanal, 1-pentanol monomer, 1-penten-3-ol, 1-propanol dimer, 1-propanol monomer, 2-heptanone, 2-pentanone, acetaldehyde and ethanol (*p* < 0.05) but significantly lower amounts of (E)-2-pentenal, 2-methyl-2-propanol, 2-methylbutanal, 3-methyl butanal, acetone, dimethyl sulfide and methyl acetate compared with subcutaneous and tail fat (*p* < 0.05). Furthermore, the proportion of 1-hydroxy-2-propanone, 2-butanone dimer and acetoin dimer was higher in tail fat, while that of 1-pentanol dimer, 2-propanol monomer, acetic acid dimer, acetic acid monomer, butanol monomer, cyclopentanone, ethyl 2-hydroxypropanoate and heptanal was lower (*p* < 0.05).

#### 3.2.3. Hierarchical Cluster Analysis

**Figure 7:**
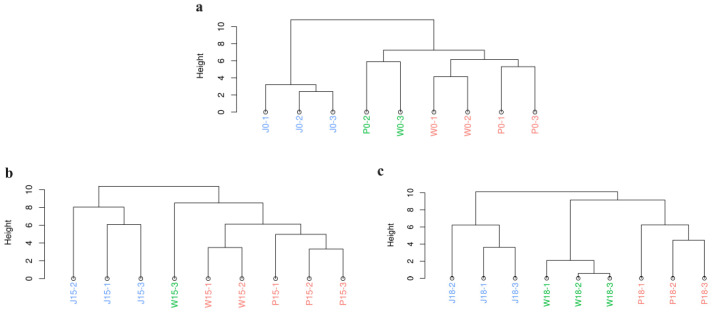
Sample Hierarchical Cluster Analysis Plot. (**a**) 0% PKM; (**b**) 15% PKM; (**c**) 18% PKM; blue for intermuscular fat, green for subcutaneous fat, red for caudal fat.

Hierarchical cluster analysis allows the aggregation of samples based on their similarity in terms of their flavor, with samples exhibiting highly similar flavors being preferentially aggregated (Figure 7). A systematic cluster analysis of the three types of adipose tissue from Tibetan sheep was therefore performed at 0%, 15% and 18% PKM levels. The clustering spectrograms showed that, at all PKM levels, subcutaneous and tail fat were grouped together, with intermuscular fat forming a separate cluster. It is speculated that there may be a link with the MUFA gradient mentioned earlier, and that the differences in VOCs between different parts of adipose tissue are also related to the depth of their location and their adaptation to temperature [21]. These findings further suggest that intermuscular fat differed significantly from subcutaneous and tail fat, irrespective of the amount of PKM added.

## 4. Correlation Analysis between Key Fatty Acids and Key Flavor Substances in Adipose Tissue of Tibetan Sheep

**Figure 8:**
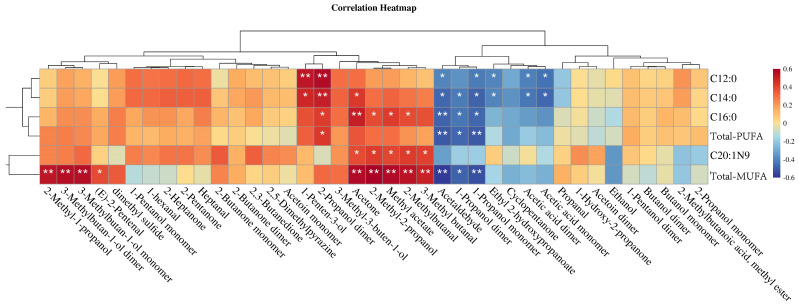
Correlation analysis chart of some fatty acids and VOCs substances, * represents significant correlation between FA and VOC (*p* < 0.05); ** represents highly significant correlation between them (*p* < 0.01).

Since fatty acid degradation generates a large number of VOCs compounds, Pearson correlation analysis was performed between selected fatty acids and VOCs compounds, with the results shown in Figure 8. Overall, C12:0 and C14:0 were positively correlated with 1-penten-3-ol, 2-propanol dimer, 3-methyl-3-buten-1-ol, acetone, acetaldehyde, 1-propanol dimer and 1-propanol monomer but negatively correlated with ethyl 2-hydroxypropanoate, acetic acid dimer and acetic acid monomer. Similarly, C16:0 was positively correlated with 2-propanol dimer, acetone, 2-methyl-2-propanol, methyl acetate and 2-methylbutanal but negatively correlated with acetaldehyde, 1-propanol dimer and 1-propanol monomer, while C24:1N9 was positively correlated with acetone, 2-methyl-2-propanol, methyl acetate, 2-methylbutanal and 3-methyl butanal. In addition, PUFA was positively correlated with 2-propanol dimer but negatively correlated with acetaldehyde, 1-propanol dimer and 1-propanol monomer, while MUFA was positively correlated with 2-methyl-1-propanol, 3-methylbutan-1-ol dimer, 3-methylbutan-1-ol monomer, acetone, 2-methyl-2-propanol, methyl acetate, 2-methylbutanal and 3-methyl butanal but negatively correlated with acetaldehyde, 1-propanol dimer and 1-propanol monomer. This indicates that unsaturated fatty acids oxidize to produce various aromatic components such as aldehydes, alcohols, esters and ketones. Similar correlations with C12:0,C14:0,C16:0 and C24:1N9 were observed for some alcohols, ketones and aldehydes in this study, which were inferred to contribute to the formation of fat flavor in Tibetan sheep, and are worthy of further investigation.

## 5. Discussion

Dietary recommendations aimed at reducing the risk of various diseases often emphasize the need to reduce the fat content of meat and adipose tissue. At the same time, when evaluating the fat content of a product, it is important to prioritize the fatty acid composition rather than examining the fat content of the meat alone [22,23]. Modifying PKM ratios provides a means of regulating lipid deposition in animal muscle and other tissues. As a result, there is better control over the fatty acid composition which ultimately enhances meat quality [24,25,26]. The results showed that C14:0, C16:0, C18:0 and C18:1N9 were the predominant fatty acids in the three types of adipose tissue, while the proportions of C12:0, C14:0, C16:0, C18:1N9, C18:2TTN6 and others were influenced by varying PKM levels. Of these, C12:0 and C14:0, which are commonly found in animal fats, are intricately linked to meat flavor and, as such, they can be used as flavor additives in the food industry [27]. Interestingly, the duration of an infant’s appetitive response was found to be longer when stimulated by myristic acid [28]. In addition, these fatty acids (C12:0 and C14:0) also possess functional properties that contribute not only to the health of the body but also to effectively inhibit the growth of *Pneumococcus*, *Streptococcus*, and *Staphylococcus aureus*, among other pathogens [29]. After adding PKM, both C12:0 and C14:0 was deposited in the three types of adipose tissue. Through the synergistic action of SCD enzymes, including FA elongases, C16:0 (both ingested and de novo synthesized) can be elongated to C18:0 before being desaturated to C18:1N9. Hence, it is not surprising that PKM addition significantly increased the proportion of C16:0, with a corresponding significant increase also observed for C18:0 and C18:1N9. These results suggest that the fatty acids in ruminant meat may be derived from direct intake (40–60%) or endogenous de novo synthesis [30,31]. The reference PKM test diet contained higher levels of C12:0, C14:0, C16:0, C18:1N9, SFA and MUFA, with a large proportion of these attributed to dietary inputs. It should be noted that C16:0 and C18:0 are important precursor substances for meat flavor. In this context, Piao et al. mentioned that MUFA and C18:1N9 were positively correlated with muscle fat content and muscle flavor, hence suggesting that a high percentage of C18:1N9 in the fat confers a higher quality flavor to the meat [32]. Moreover, in agreement with most previous findings, C18:1N9 is not only a major component of meat MUFA, but it also enhances immunity while having a favorable effect on the prevention of cancer and inflammatory diseases [33]. Furthermore, the addition of PKM was accompanied by increased amounts of MUFA, PUFA, n-3, n-6 and SFA. In this case, while an increase in SFA (including C12:0, C14:0, and C16:0) is often viewed negatively from a nutritional perspective, studies have shown that, through heat decomposition during the cooking process, each fatty acid may yield specific oxides that contribute in some way to meat flavor [34]. Dietary changes showed that high ratios of N6/N3 were more likely to predispose to cardiovascular and chronic inflammatory diseases, but both N3 and N6 polyunsaturated fatty acids were protective against both cardiovascular system and diabetic complications [35,36]. Therefore, it was concluded that adding PKM to the diet positively influenced the fatty acid content and composition of Tibetan sheep fat, but further investigation on its decomposition during storage and cooking is warranted.

Subcutaneous adipose tissue, accounting for at least 70% of the body fat, represents an important source of energy and insulation in animals [37]. Specifically, tail fat is that part of the animal which stores energy to adapt to food shortages. However, consumers believe that it has a special flavor that enhances the taste and tenderness of meat products [9]. Intermuscular fat, on the other hand, is a peculiar type of adipose tissue in which the content, reflected in the marbling score, is closely related to the flavor, tenderness and juiciness of pork, all of which are important indicators of meat quality [38]. Differences in fatty acids between the three types of adipose tissue were mainly observed for C15:1N5 and C18:1N9, but were dispelled by the addition of PKM to the diets. These results suggest that the fatty acids in animal fats were positively correlated with those present in the ingested diets [39]. Subcutaneous and intermuscular fat had the highest total SFA content, followed by MUFA and PUFA, with similar results reported by Güler [9]. However, tail fat had a higher total MUFA content compared with SFA, possibly due to its higher amount of C18:1N9. Some studies have reported that the internal adipose tissue of sheep was more saturated than its subcutaneous fat, although this was in contradiction with the current results. In fact, previous studies did found that the saturated fat content was consistently lower in the intermuscular fat of Tibetan sheep [21]. Differences between adipose layers may arise due to their adaptation to temperature in order to maintain the physical mobility of lipids in the tissues [21]. In this context, a gradient difference in MUFA content has been reported between the following adipose tissues: subcutaneous fat > intermuscular fat > flare fat [40]. In the present study, a similar gradient in MUFA content was observed, with the highest amount in tail fat, followed by subcutaneous and intermuscular fat. Thus, the content seemed to be related to the location depth of the fat.

Flavor plays a pivotal role in consumer preference for meat, with the amount and composition of fat in the meat being also crucial determinants of flavor, texture and overall sensory attributes. Fat is a major source of numerous aroma-active compounds that interact with the Merad reaction pathway to influence the formation of aroma molecules [41]. Therefore, to better understand the impact of PKM on Tibetan sheep muscle, it is important to evaluate the flavor profile of lipids as solvents for VOCs substances [42]. This study revealed that the addition of PKM increased the proportion of ketones but decreased the proportion of alcohols and acids in the subcutaneous and tail fat of Tibetan sheep, with maximum effects observed at 18% PKM. Conversely, the proportion of aldehydes was lower in tail and intermuscular fat. Ketones, produced by lipid oxidation, are associated with a creamy, fruity and spicy flavor at high concentrations [43]. In contrast, compounds such as acetoin monomer and 2,3-butanedione may contribute a buttery flavor, although the latter, which is a product of the Meladic reaction, may have a lower odor threshold [44]. Similarly, acetone, which is converted from acetyl coenzyme A produced during glycogen degradation, imparts a buttery and fruity flavor to meat [45], while 2-butanone, which is produced by the decarboxylation of β-keto acids or the β-oxidation of saturated fatty acids, may serve as a precursor for the formation of fat flavor [46]. And the proportions of these substances were significantly higher in both subcutaneous and tail fat, and the proportion of acetone in intermuscular fat was also significantly higher after the addition of PKM. Overall, the addition of PKM significantly increased the proportion of these substances in both subcutaneous and tail fat, with intermuscular fat also displaying a significantly higher proportion of acetone.

Alcohols, primarily ethanol-based and produced through the oxidation and breakdown of polyunsaturated fatty acids, constitute a significant proportion of Tibetan sheep adipose tissue, and they are usually associated with a relatively mild fruity or vegetal aroma and sweet flavors [47]. However, while alcohols generally have a higher threshold and have minimal impact on volatile meat flavors, they still contribute synergistically to overall flavor [48]. Marta uses ethanol as a volatile spoilage marker for meat, which produces undesirable flavors [49]. After adding PKM, the ethanol content decreased significantly in subcutaneous and tail fat but increased in intermuscular fat, especially at 15% PKM. In livestock such as cattle and sheep, specific *Clostridium thermophilum* can secrete cellulase that breaks down the cellulose in food into individual glucose molecules which can, in turn, be converted into ethanol through yeast fermentation [50,51]. With PKM being rich in cellulose, the increase in ethanol content that normally occurs in intermuscular fat [52] could be attributed to the prevalence of the thermophilic bacteria *Thermus thermophilus* in the muscle. One of the most important aroma substances in meat is 1-penten-3-ol which has a mushroom odor [53], and in the current study, adding PKM increased the amount of this compound. On the other hand, while aldehydes are also important contributors of pleasant grassy and fatty flavors [54], a small amount decrease in their content in tail and intermuscular fat at higher PKM levels. Acids are generally derived from lipid oxidation, with some branched-chain ones usually associated with lamb and sweat properties. Hence, in excessive concentrations, they produce an undesirable odor and taste [3,55]. However, interestingly, the addition of PKM decreased the proportion of acids in the fat. Esters are mainly produced from alcohols and organic acids through non-enzymatic and microbial enzymatic esterification, and they not only impart fruity and sweet flavors to meat, but also help to mask fatty flavors [54,56]. In this study, the amount of esters was slightly lower mainly due to a decrease in alcohols and acids. Finally, pyrazines, associated with characteristic roasted meat flavors [57], increased in content with the addition of PKM, especially in the case of 2,5-dimethylpyrazine.

In summary, the incorporation of PKM in the diets of Tibetan sheep had favorable effects on fat composition. Previous studies have also highlighted that the influence of PKM on meat flavor, hence paving the way for future exploration of the impact of palm meal on the flavor of animal meat.

## 6. Conclusions

Increasing PKM levels in the diet of Tibetan sheep induced significant changes in the fatty acid content and composition of different parts of their adipose tissues. Some of them could be deemed advantageous for human health while others could be considered adverse. Based on the fat composition and content, differences were evident between the three types of adipose tissue, i.e., subcutaneous, tail and intermuscular fats. Overall, the addition of PKM favored an improved flavor profile for Tibetan sheep fat.

## Figures and Tables

**Table 1:** Composition and Nutritional Level of Concentrate Supplements in Diets of sheep.

Raw Material Composition/%	ZL-0	ZL-15	ZL-18
Maize	65.5	54.20	51.80
Soybean meal	8.00	5.00	5.00
Rapeseed meal	16.00	15.00	15.00
Cottonseed meal	4.00	4.30	3.70
PKM	0.00	15.00	18.00
Common salt	0.50	0.50	0.50
Mountain flour	1.00	1.00	1.00
premix	5.00	5.00	5.00
Nutritional Level/%			
Crude protein	15.15	15.12	15.13
Crude fat	2.70	3.42	3.58
NDF	3.83	5.78	6.16
Lysine	0.66	0.63	0.63
Methionine	0.26	0.28	0.28
Calcium	0.54	0.52	0.51
Phosphorus	0.43	0.38	0.36

Nutritional composition of PKM: 7.90% moisture, 4.60% crude ash, 16.50% crude protein, 6.00% crude fat, 33.20% acid detergent fiber, 72.20% neutral detergent fiber.

**Table 2:** Composition and content of fatty acids in PKM (ug/g).

FA Composition		FA Composition	
C10:0	1261.82 ± 173.44	C18:3N3	3.39 ± 0.20
C11:0	17.46 ± 0.67	C20:0	51.01 ± 2.05
C12:0	9787.25 ± 162.37	C20:1N9	54.41 ± 1.30
C13:0	24.87 ± 0.76	C20:5N3	7.03 ± 0.19
C14:0	5041.93 ± 98.38	C22:1N9	31.1 ± 4.91
C15:0	6.11 ± 0.25	C22:2N6	6.83 ± 1.02
C15:1N5	11.11 ± 0.34	C22:4N6	12.89 ± 3.70
C16:0	4021.43 ± 102.70	C23:0	7.53 ± 0.21
C16:1N7	8.92 ± 0.09	C24:0	32.99 ± 0.58
C17:0	9.13 ± 0.25	C6:0	4.1 ± 1.84
C17:1N7	4.08 ± 0.08	C8:0	646.9 ± 384.59
C18:0	1202.19 ± 48.25	MUFA	6434.71 ± 89.54
C18:1N9	6310.75 ± 91.79	PUFA	1174.6 ± 34.22
C18:1TN9	12.79 ± 0.16	SFA	22,116.38 ± 289.32
C18:2N6	1137.45 ± 36.91	N-3	11.21 ± 0.22
C18:2TTN6	2.04 ± 0.21	N-6	1163.4 ± 34.12

The data was expressed as mean ± standard deviation (n = 3). SFA, MUFA and PUFA: Saturated, monounsaturated and polyunsaturated fatty acids. N-3 and N-6: Ω3 series unsaturated fatty acids and Ω3 series unsaturated fatty acids.

**Table 3:** Effects of three PKM levels on the composition and content of FAs in subcutaneous fat, tail fat and intermuscular fat of Tibetan sheep (μg/10 g).

Item FAs	Diet ^1^			SEM	*p*-Value
	0%	15%	18%		
subcutaneous fat	P0	P15	P18		
SFA	7439.14 ± 547.83	8312.96 ± 735.55	9139.31 ± 897.07	604.77	0.080
MUFA	6137.44 ± 848.52	7749.43 ± 940.27	8298.47 ± 1853.62	1058.30	0.186
PUFA	401.36 ± 78.44	496.05 ± 37.33	495.01 ± 139.11	77.31	0.425
N-3	40.78 ± 10.20	63.79 ± 10.21	54.66 ± 18.54	11.07	0.193
N-6	360.58 ± 68.61	432.26 ± 27.18	440.35 ± 120.62	66.66	0.467
N-6/N-3	8.93 ± 0.72 ^a^	6.85 ± 0.66 ^b^	8.17 ± 0.52 ^ab^	0.53	0.021
tail fat	W0	W15	W18		
SFA	7115.19 ± 260.12	7320.24 ± 987.87	8206.38 ± 565.59	550.44	0.190
MUFA	7844.32 ± 1184.21	6740.95 ± 2078.44	9867.08 ± 1991.05	1467.16	0.178
PUFA	375.24 ± 74.34	427.97 ± 71.88	509.22 ± 41.35	52.50	0.108
N-3	39.34 ± 8.35 ^b^	49.28 ± 7.71 ^ab^	59.26 ± 4.14 ^a^	5.70	0.036
N-6	335.90 ± 66.03	378.69 ± 64.81	449.96 ± 37.33	47.03	0.125
N-6/N-3	8.56 ± 0.22 ^a^	7.69 ± 0.51 ^b^	7.59 ± 0.18 ^b^	0.28	0.023
intermuscular fat	J0	J15	J18		
SFA	4819.11 ± 2797.93	7074.86 ± 2809.28	8639.12 ± 2471.87	2225.72	0.298
MUFA	4223.10 ± 1703.64	5463.27 ± 1774.92	6859.68 ± 1572.00	1376.30	0.239
PUFA	238.87 ± 134.95	357.97 ± 148.53	498.72 ± 283.26	163.64	0.348
N3	17.93 ± 14.32	40.37 ± 20.96	55.05 ± 36.94	21.13	0.284
N6	220.94 ± 120.75	317.60 ± 127.59	443.67 ± 246.45	142.67	0.358
N6/N3	14.30 ± 4.66	8.48 ± 1.78	9.12 ± 2.61	2.66	0.132

Diet ^1^: Different levels of PKM addition to diets, 0%, 15, 18%. SFA, MUFA and PUFA: Saturated, monounsaturated and polyunsaturated fatty acids. N-3 and n-6: Ω3 series unsaturated fatty acids and Ω3 series unsaturated fatty acids. P: subcutaneous fat, W: tail fat, J; intermuscular fat; lowercase letters: the same lowercase letters or no letters indicate no significant difference (*p* > 0.05), and different lowercase letters indicate significant difference (*p* < 0.05), the following tables are all the same.

**Table 4:** Effects of the composition and content of FAs among different parts of adipose tissue of Tibetan sheep under the same PKM level (μg/10 g).

Item FAs	Adipose Tissue			SEM	*p*-Value
	Subcutaneous Fat	Tail Fat	Intermuscular Fat		
0% PKM	P0	W0	J0		
SFA	7439.14 ± 547.83	7115.19 ± 260.12	4819.11 ± 2797.93	1349.59	0.188
MUFA	6137.44 ± 848.52 ^ab^	7844.32 ± 1184.21 ^a^	4223.10 ± 1703.64 ^b^	1056.70	0.039
PUFA	401.36 ± 78.44	375.24 ± 74.34	238.87 ± 134.95	81.50	0.182
N-3	40.78 ± 10.20	39.34 ± 8.35	17.93 ± 14.32	9.18	0.083
N-6	360.58 ± 68.61	335.90 ± 66.03	220.94 ± 120.75	72.49	0.202
N-6/N-3	8.93 ± 0.72	8.56 ± 0.22	14.30 ± 4.66	2.23	0.073
15% PKM	P15	W15	J15		
SFA	8312.96 ± 735.55	7320.24 ± 987.87	7074.86 ± 2809.28	1481.04	0.692
MUFA	7749.43 ± 940.27	6740.95 ± 2078.44	5463.27 ± 1774.92	1362.54	0.314
PUFA	496.05 ± 37.33	427.97 ± 71.88	357.97 ± 148.53	79.75	0.297
N-3	63.79 ± 10.21	49.28 ± 7.71	40.37 ± 20.96	11.58	0.205
N-6	432.26 ± 27.18	378.69 ± 64.81	317.60 ± 127.59	68.67	0.318
N-6/N-3	6.85 ± 0.66	7.69 ± 0.51	8.48 ± 1.78	0.93	0.290
18% PKM	P18	W18	J18		
SFA	9139.31 ± 897.07	8206.38 ± 565.59	8639.12 ± 2471.87	1267.96	0.771
MUFA	8298.47 ± 1853.62	9867.08 ± 1991.05	6859.68 ± 1572.00	1481.09	0.208
PUFA	495.01 ± 139.11	509.22 ± 41.35	498.72 ± 283.26	150.04	0.995
N-3	54.66 ± 18.54	59.26 ± 4.14	55.05 ± 36.94	19.58	0.967
N-6	440.35 ± 120.62	449.96 ± 37.33	443.67 ± 246.45	130.54	0.997
N-6/N-3	8.17 ± 0.52	7.59 ± 0.18	9.12 ± 2.61	1.26	0.512

SFA, MUFA and PUFA: Saturated, monounsaturated and polyunsaturated fatty acids. N-3 and n-6: Ω3 series unsaturated fatty acids and Ω3 series unsaturated fatty acids. P: subcutaneous fat, W: tail fat, J; intermuscular fat. Lowercase letters: the same lowercase letters or no letters indicate no significant difference (*p* > 0.05), and different lowercase letters indicate significant difference (*p* < 0.05).

**Table 5:** Effects of three PKM levels on the composition and content of VOCs in subcutaneous fat, tail fat and intermuscular fat of Tibetan sheep (%).

FCs	Subcutaneous Fat			*p*-Value	Tail Fat			*p*-Value	Intermuscular Fat			*p*-Value
	P0	P15	P18		W0	W15	W18		J0	J15	J18	
(E)-2-Pentenal	0.34 ± 0.11 ^b^	0.96 ± 0.15 ^a^	1.42 ± 0.37 ^a^	0.004	1.75 ± 0. 1 ^a^	1.15 ± 0.1 ^b^	0.82 ± 0.04 ^c^	0.000	0.54 ± 0.08 ^a^	0.31 ± 0.06 ^b^	0.34 ± 0.02 ^b^	0.005
1-hexanal	0.27 ± 0.05	0.30 ± 0.03	0.33 ± 0.01	0.255	0.28 ± 0.04 ^a^	0.24 ± 0.04 ^ab^	0.16 ± 0.02 ^b^	0.015	0.29 ± 0.03 ^b^	0.21 ± 0.03 ^b^	0.72 ± 0.08 ^a^	0.000
1-Hydroxy-2-propanone	0.16 ± 0.06 ^b^	0.19 ± 0.04 ^ab^	0.33 ± 0.08 ^a^	0.034	0.15 ± 0.08 ^b^	0.18 ± 0.07 ^b^	0.6 ± 0.03 ^a^	0.000	0.7 ± 0.07 ^a^	0.17 ± 0.07 ^c^	0.42 ± 0.11 ^b^	0.001
1-Pentanol dimer	0.03 ± 0.02	0.04 ± 0.00	0.04 ± 0.01	0.695	0.03 ± 0.02	0.04 ± 0.02	0.01 ± 0.01	0.181	0.04 ± 0	0.03 ± 0.01	0.04 ± 0.01	0.300
1-Pentanol monomer	0.14 ± 0.05	0.18 ± 0.00	0.15 ± 0.02	0.345	0.14 ± 0.05	0.14 ± 0.05	0.17 ± 0.04	0.689	0.32 ± 0.05 ^b^	0.13 ± 0.03 ^c^	0.75 ± 0.08 ^a^	0.000
1-Penten-3-ol	0.65 ± 0.03 ^b^	0.89 ± 0.07 ^a^	0.74 ± 0.03 ^b^	0.003	0.65 ± 0.03 ^a^	0.66 ± 0.01 ^a^	0.55 ± 0.03 ^b^	0.003	0.59 ± 0.02 ^b^	0.55 ± 0.04 ^b^	0.83 ± 0.04 ^a^	0.000
1-Propanol dimer	0.52 ± 0.00 ^a^	0.24 ± 0.02 ^c^	0.31 ± 0.03 ^b^	0.000	0.71 ± 0.26	0.37 ± 0.03	0.5 ± 0.01	0.092	3.68 ± 0.62 ^a^	0.75 ± 0.03 ^b^	0.96 ± 0.13 ^b^	0.000
1-Propanol monomer	2.63 ± 0.03 ^a^	1.42 ± 0.15 ^c^	1.79 ± 0.19 ^b^	0.000	3.08 ± 0.57 ^a^	2.27 ± 0.14 ^ab^	2.12 ± 0.02 ^b^	0.028	6.09 ± 0.49 ^a^	2.99 ± 0.08 ^b^	3.32 ± 0.26 ^b^	0.000
2,3-Butanedione	0.51 ± 0.07 ^b^	0.49 ± 0.05 ^b^	2.59 ± 0.85 ^a^	0.003	0.42 ± 0.04 ^c^	0.57 ± 0.08 ^b^	2.00 ± 0.01 ^a^	0.000	2.80 ± 0.66 ^a^	1.16 ± 0.02 ^b^	2.47 ± 0.49 ^a^	0.012
2,5-Dimethylpyrazine	0.14 ± 0.06 ^b^	0.21 ± 0.04 ^b^	0.41 ± 0.10 ^a^	0.008	0.12 ± 0.04 ^b^	0.15 ± 0.07 ^b^	0.56 ± 0.02 ^a^	0.000	0.64 ± 0.02 ^a^	0.21 ± 0.07 ^b^	0.51 ± 0.10 ^a^	0.001
2-Butanone dimer	0.50 ± 0.01	0.49 ± 0.11	0.55 ± 0.04	0.547	0.47 ± 0.04 ^b^	0.48 ± 0.03 ^b^	1.78 ± 0.01 ^a^	0.000	0.96 ± 0.07 ^b^	1.04 ± 0.10 ^b^	1.24 ± 0.06 ^a^	0.012
2-Butanone monomer	1.05 ± 0.02 ^b^	0.99 ± 0.05 ^b^	1.32 ± 0.08 ^a^	0.001	1.15 ± 0.1 ^a^	1.14 ± 0.03 ^b^	1.34 ± 0.02 ^a^	0.013	1.16 ± 0.04	1.1 ± 0.13	1.22 ± 0.09	0.335
2-Heptanone	0.05 ± 0.02	0.07 ± 0.01	0.10 ± 0.02	0.067	0.06 ± 0.02	0.07 ± 0.02	0.04 ± 0.01	0.273	0.09 ± 0.00 ^b^	0.06 ± 0.01 ^b^	0.16 ± 0.05 ^a^	0.012
2-Methyl-1-propanol	0.76 ± 0.05 ^a^	0.52 ± 0.04 ^b^	0.53 ± 0.03 ^b^	0.000	0.64 ± 0.05 ^b^	0.70 ± 0.04 ^b^	1.48 ± 0.02 ^a^	0.000	0.39 ± 0.01	0.47 ± 0.05	0.39 ± 0.01	0.037
2-Methyl-2-propanol	0.38 ± 0.03 ^b^	0.43 ± 0.05 ^b^	1.90 ± 0.40 ^a^	0.000	0.36 ± 0.04 ^b^	0.44 ± 0.03 ^b^	2.09 ± 0.03 ^a^	0.000	0.28 ± 0.03	0.35 ± 0.05	0.33 ± 0.00	0.138
2-Methylbutanal	0.04 ± 0.01 ^b^	0.04 ± 0.00 ^b^	0.70 ± 0.41 ^a^	0.023	0.03 ± 0.01 ^b^	0.05 ± 0.01 ^b^	0.56 ± 0.01 ^a^	0.000	0.03 ± 0.00	0.04 ± 0.01	0.05 ± 0.02	0.444
2-Methylbutanoic acid, methyl ester	0.61 ± 0.05 ^a^	0.63 ± 0.03 ^a^	0.40 ± 0.03 ^b^	0.000	0.43 ± 0.02 ^b^	0.49 ± 0.03 ^a^	0.41 ± 0.01 ^b^	0.012	0.33 ± 0.03 ^c^	0.88 ± 0.01 ^a^	0.62 ± 0.15 ^b^	0.001
2-Pentanone	0.04 ± 0.02 ^b^	0.05 ± 0.01 ^ab^	0.07 ± 0.00 ^a^	0.048	0.06 ± 0.01 ^b^	0.05 ± 0.02 ^b^	0.12 ± 0.01 ^a^	0.002	0.15 ± 0.01	0.09 ± 0.03	0.44 ± 0.24	0.046
2-Propanol dimer	0.84 ± 0.06 ^b^	1.26 ± 0.11 ^a^	0.92 ± 0.19 ^b^	0.019	0.6 ± 0.02 ^b^	0.94 ± 0.05 ^a^	1.00 ± 0.03 ^a^	0.000	0.44 ± 0.01 ^b^	0.93 ± 0.05 ^a^	0.83 ± 0.05 ^a^	0.000
2-Propanol monomer	1.31 ± 0.02	1.60 ± 0.09	1.43 ± 0.29	0.215	1.33 ± 0.08 ^b^	1.69 ± 0.05 ^a^	0.71 ± 0.01 ^c^	0.000	0.99 ± 0.05	1.06 ± 0.13	1.05 ± 0.09	0.702
3-Methyl butanal	0.06 ± 0.01 ^b^	0.05 ± 0.00 ^b^	0.58 ± 0.32 ^a^	0.021	0.07 ± 0.01 ^b^	0.06 ± 0.01 ^b^	0.33 ± 0.01 ^a^	0.000	0.05 ± 0.00 ^a^	0.06 ± 0.01 ^a^	0.04 ± 0.00 ^b^	0.021
3-Methyl-3-buten-1-ol	0.05 ± 0.02 ^b^	0.07 ± 0.00 ^ab^	0.10 ± 0.01 ^a^	0.009	0.05 ± 0.02 ^b^	0.06 ± 0.02 ^b^	0.13 ± 0.00 ^a^	0.001	0.08 ± 0.00	0.09 ± 0.01	0.1 ± 0.01	0.230
3-Methylbutan-1-ol dimer	0.12 ± 0.06	0.17 ± 0.02	0.19 ± 0.02	0.133	0.15 ± 0.07 ^b^	0.14 ± 0.07 ^b^	0.79 ± 0.01 ^a^	0.000	0.13 ± 0.01	0.12 ± 0.06	0.13 ± 0.02	0.877
3-Methylbutan-1-ol monomer	0.66 ± 0.21	0.46 ± 0.06	0.88 ± 0.20	0.061	0.52 ± 0.15 ^b^	0.39 ± 0.13 ^b^	3.52 ± 0.03 ^a^	0.000	0.43 ± 0.02	0.34 ± 0.11	0.46 ± 0.09	0.252
Acetaldehyde	2.40 ± 0.12 ^a^	2.23 ± 0.16 ^a^	1.62 ± 0.08 ^b^	0.001	3.79 ± 0.13 ^a^	3.09 ± 0.28 ^b^	1.11 ± 0.08 ^c^	0.000	5.48 ± 0.36 ^a^	2.77 ± 0.25 ^b^	3.59 ± 0.63 ^b^	0.001
Acetic acid dimer	1.54 ± 0.48 ^a^	0.71 ± 0.04 ^b^	0.52 ± 0.07 ^b^	0.010	1.01 ± 0.12 ^a^	0.63 ± 0.16 ^b^	0.27 ± 0.07 ^c^	0.001	0.83 ± 0.03 ^a^	0.53 ± 0.11 ^b^	0.48 ± 0.07 ^b^	0.003
Acetic acid monomer	11.39 ± 1.34 ^a^	7.43 ± 0.30 ^b^	6.50 ± 0.52 ^b^	0.001	9.8 ± 0.73 ^a^	7.54 ± 0.59 ^b^	3.88 ± 0.04 ^c^	0.000	7.30 ± 0.35 ^a^	6.47 ± 0.92 ^ab^	5.12 ± 0.35 ^b^	0.012
Acetoin dimer	0.35 ± 0.11 ^b^	0.47 ± 0.09 ^b^	1.17 ± 0.42 ^a^	0.017	0.36 ± 0.1 ^b^	0.37 ± 0.13 ^b^	2.53 ± 0.06 ^a^	0.000	3.35 ± 0.29 ^a^	0.54 ± 0.21 ^b^	1.41 ± 0.59 ^b^	0.000
Acetoin monomer	3.19 ± 0.26 ^b^	4.32 ± 1.05 ^b^	9.27 ± 2.05 ^a^	0.003	2.69 ± 0.09 ^c^	3.46 ± 0.08 ^b^	9.74 ± 0.09 ^a^	0.000	13.03 ± 0.60 ^a^	5.99 ± 1.05 ^b^	8.48 ± 1.98 ^b^	0.002
Acetone	14.55 ± 1.26 ^b^	16.13 ± 0.70 ^b^	26.08 ± 1.78 ^a^	0.000	16.86 ± 1.48 ^b^	17.07 ± 0.42 ^b^	21.28 ± 0.28 ^a^	0.002	11.44 ± 0.33 ^b^	15.96 ± 1.78 ^a^	16.5 ± 1.96 ^a^	0.013
Butanol dimer	0.25 ± 0.03	0.18 ± 0.04	0.20 ± 0.02	0.069	0.23 ± 0.04	0.18 ± 0.03	0.17 ± 0.02	0.127	0.11 ± 0.01 ^b^	0.39 ± 0.14 ^a^	0.23 ± 0.06 ^ab^	0.023
Butanol monomer	2.29 ± 0.08	1.99 ± 0.21	2.18 ± 0.17	0.148	2.25 ± 0.01 ^a^	2.17 ± 0.06 ^a^	1.58 ± 0.03 ^b^	0.000	1.19 ± 0.01 ^b^	2.75 ± 0.42 ^a^	2.00 ± 0.37 ^a^	0.003
Cyclopentanone	0.07 ± 0.02 ^b^	0.09 ± 0.01 ^ab^	0.13 ± 0.02 ^a^	0.028	0.11 ± 0.02	0.09 ± 0.01	0.07 ± 0.01	0.059	0.22 ± 0.02 ^a^	0.06 ± 0.02 ^b^	0.07 ± 0.00 ^b^	0.000
dimethyl sulfide	0.77 ± 0.03 ^b^	1.58 ± 0.22 ^a^	1.75 ± 0.21 ^a^	0.001	1.41 ± 0.01 ^b^	1.61 ± 0.04 ^a^	0.84 ± 0.00 ^c^	0.000	0.54 ± 0.02 ^b^	0.70 ± 0.03 ^a^	0.52 ± 0.01 ^b^	0.000
Ethanol	33.61 ± 1.86 ^a^	32.15 ± 1.26 ^a^	15.30 ± 2.79 ^b^	0.000	26.66 ± 1.16 ^a^	27.73 ± 1.81 ^a^	18.26 ± 0.11 ^b^	0.000	21.25 ± 0.74 ^c^	36.21 ± 0.55 ^a^	30.53 ± 2.64 ^b^	0.000
Ethyl 2-hydroxypropanoate	1.31 ± 0.49 ^a^	0.51 ± 0.06 ^b^	0.48 ± 0.03 ^b^	0.019	0.82 ± 0.09 ^a^	0.46 ± 0.11 ^b^	0.17 ± 0.06 ^c^	0.000	0.53 ± 0.03 ^a^	0.31 ± 0.11 ^b^	0.34 ± 0.02 ^b^	0.012
Heptanal	0.27 ± 0.05	0.28 ± 0.03	0.31 ± 0.02	0.458	0.26 ± 0.01 ^a^	0.26 ± 0.02 ^a^	0.16 ± 0.02 ^b^	0.000	0.22 ± 0.01 ^b^	0.21 ± 0.03 ^b^	0.46 ± 0.01 ^a^	0.000
Methyl acetate	0.04 ± 0.01 ^b^	0.05 ± 0.01 ^b^	0.18 ± 0.03 ^a^	0.000	0.05 ± 0.01 ^b^	0.07 ± 0.01 ^b^	0.13 ± 0.01 ^a^	0.000	0.03 ± 0.01	0.03 ± 0.00	0.04 ± 0.01	0.060
Propanal	3.35 ± 0.81	2.28 ± 0.59	2.29 ± 0.58	0.157	2.96 ± 0.24 ^a^	1.85 ± 0.27 ^b^	1.24 ± 0.06 ^c^	0.000	0.58 ± 0.07 ^b^	1.07 ± 0.33 ^a^	0.11 ± 0.01 ^b^	0.003

Lowercase letters: the same lowercase letters or no letters indicate no significant difference (*p* > 0.05), and different lowercase letters indicate significant difference (*p* < 0.05).

**Table 6:** Differences in composition and content of VOCs among different parts of adipose tissue of Tibetan sheep at the same PKM level (%).

FCs	0%			*p*-Value	18%			*p*-Value	21%			*p*-Value
	P0	W0	J0		P15	W15	J15		P18	W18	J18	
(E)-2-Pentenal	0.34 ± 0.11 ^b^	1.75 ± 0.1 ^a^	0.54 ± 0.08 ^b^	0.000	0.96 ± 0.15 ^a^	1.15 ± 0.1 ^a^	0.31 ± 0.06 ^b^	0.000	1.42 ± 0.37 ^a^	0.82 ± 0.04 ^b^	0.34 ± 0.02 ^b^	0.002
1-hexanal	0.27 ± 0.05	0.28 ± 0.04	0.29 ± 0.03	0.808	0.30 ± 0.03 ^a^	0.24 ± 0.04 ^ab^	0.21 ± 0.03 ^b^	0.039	0.33 ± 0.01 ^b^	0.16 ± 0.02 ^c^	0.72 ± 0.08 ^a^	0.000
1-Hydroxy-2-propanone	0.16 ± 0.06 ^b^	0.15 ± 0.08 ^b^	0.70 ± 0.07 ^a^	0.000	0.19 ± 0.04	0.18 ± 0.07	0.17 ± 0.07	0.893	0.33 ± 0.08 ^b^	0.6 ± 0.03 ^a^	0.42 ± 0.11 ^ab^	0.014
1-Pentanol dimer	0.03 ± 0.02	0.03 ± 0.02	0.04 ± 0.00	0.964	0.04 ± 0.00	0.04 ± 0.02	0.03 ± 0.01	0.498	0.04 ± 0.01 ^a^	0.01 ± 0.01 ^b^	0.04 ± 0.01 ^a^	0.010
1-Pentanol monomer	0.14 ± 0.05 ^b^	0.14 ± 0.05 ^b^	0.32 ± 0.05 ^a^	0.005	0.18 ± 0.00	0.14 ± 0.05	0.13 ± 0.03	0.283	0.15 ± 0.02 ^b^	0.17 ± 0.04 ^b^	0.75 ± 0.08 ^a^	0.000
1-Penten-3-ol	0.65 ± 0.03	0.65 ± 0.03	0.59 ± 0.02	0.088	0.89 ± 0.07 ^a^	0.66 ± 0.01 ^b^	0.55 ± 0.04 ^b^	0.000	0.74 ± 0.03 ^b^	0.55 ± 0.03 ^c^	0.83 ± 0.04 ^a^	0.000
1-Propanol dimer	0.52 ± 0.00 ^b^	0.71 ± 0.26 ^b^	3.68 ± 0.62 ^a^	0.000	0.24 ± 0.02 ^c^	0.37 ± 0.03 ^b^	0.75 ± 0.03 ^a^	0.000	0.31 ± 0.03 ^c^	0.5 ± 0.01 ^b^	0.96 ± 0.13 ^a^	0.000
1-Propanol monomer	2.63 ± 0.03 ^b^	3.08 ± 0.57 ^b^	6.09 ± 0.49 ^a^	0.000	1.42 ± 0.15 ^c^	2.27 ± 0.14 ^b^	2.99 ± 0.08 ^a^	0.000	1.79 ± 0.19 ^b^	2.12 ± 0.02 ^b^	3.32 ± 0.26 ^a^	0.000
2,3-butanedione	0.51 ± 0.07 ^b^	0.42 ± 0.04 ^b^	2.80 ± 0.66 ^a^	0.000	0.49 ± 0.05 ^b^	0.57 ± 0.08 ^b^	1.16 ± 0.02 ^a^	0.000	2.59 ± 0.85	2.00 ± 0.01	2.47 ± 0.49	0.449
2,5-Dimethylpyrazine	0.14 ± 0.06 ^b^	0.12 ± 0.04 ^b^	0.64 ± 0.02 ^a^	0.000	0.21 ± 0.04	0.15 ± 0.07	0.21 ± 0.07	0.457	0.41 ± 0.10	0.56 ± 0.02	0.51 ± 0.10	0.175
2-Butanone dimer	0.50 ± 0.01 ^b^	0.47 ± 0.04 ^b^	0.96 ± 0.07 ^a^	0.000	0.49 ± 0.11 ^b^	0.48 ± 0.03 ^b^	1.04 ± 0.10 ^a^	0.000	0.55 ± 0.04 ^c^	1.78 ± 0.01 ^a^	1.24 ± 0.06 ^b^	0.000
2-Butanone monomer	1.05 ± 0.02	1.15 ± 0.10	1.16 ± 0.04	0.137	0.99 ± 0.05	1.14 ± 0.03	1.10 ± 0.13	0.126	1.32 ± 0.08	1.34 ± 0.02	1.22 ± 0.09	0.184
2-Heptanone	0.05 ± 0.02	0.06 ± 0.02	0.09 ± 0.00	0.092	0.07 ± 0.01	0.07 ± 0.02	0.06 ± 0.01	0.880	0.10 ± 0.02 ^ab^	0.04 ± 0.01 ^b^	0.16 ± 0.05 ^a^	0.007
2-Methyl-1-propanol	0.76 ± 0.05 ^a^	0.64 ± 0.05 ^b^	0.39 ± 0.01 ^c^	0.000	0.52 ± 0.04 ^b^	0.70 ± 0.04 ^a^	0.47 ± 0.05 ^b^	0.001	0.53 ± 0.03 ^b^	1.48 ± 0.02 ^a^	0.39 ± 0.01 ^c^	0.000
2-Methyl-2-propanol	0.38 ± 0.03 ^a^	0.36 ± 0.04 ^ab^	0.28 ± 0.03 ^b^	0.035	0.43 ± 0.05	0.44 ± 0.03	0.35 ± 0.05	0.071	1.90 ± 0.40 ^a^	2.09 ± 0.03 ^a^	0.33 ± 0.00 ^b^	0.000
2-Methylbutanal	0.04 ± 0.01	0.03 ± 0.01	0.03 ± 0.00	0.475	0.04 ± 0.00	0.05 ± 0.01	0.04 ± 0.01	0.245	0.70 ± 0.41 ^a^	0.56 ± 0.01 ^ab^	0.05 ± 0.02 ^b^	0.035
2-Methylbutanoic acid, methyl ester	0.61 ± 0.05 ^a^	0.43 ± 0.02 ^b^	0.33 ± 0.03 ^c^	0.000	0.63 ± 0.03 ^b^	0.49 ± 0.03 ^c^	0.88 ± 0.01 ^a^	0.000	0.40 ± 0.03	0.41 ± 0.01	0.62 ± 0.15	0.043
2-Pentanone	0.04 ± 0.02 ^b^	0.06 ± 0.01 ^b^	0.15 ± 0.01 ^a^	0.000	0.05 ± 0.01	0.05 ± 0.02	0.09 ± 0.03	0.046	0.07 ± 0.00 ^b^	0.12 ± 0.01 ^ab^	0.44 ± 0.24 ^a^	0.035
2-Propanol dimer	0.84 ± 0.06 ^a^	0.60 ± 0.02 ^b^	0.44 ± 0.01 ^c^	0.000	1.26 ± 0.11 ^a^	0.94 ± 0.05 ^b^	0.93 ± 0.05 ^b^	0.004	0.92 ± 0.19	1.00 ± 0.03	0.83 ± 0.05	0.302
2-Propanol monomer	1.31 ± 0.02 ^a^	1.33 ± 0.08 ^a^	0.99 ± 0.05 ^b^	0.001	1.60 ± 0.09 ^a^	1.69 ± 0.05 ^a^	1.06 ± 0.13 ^b^	0.000	1.43 ± 0.29 ^a^	0.71 ± 0.01 ^b^	1.05 ± 0.09 ^ab^	0.007
3-Methyl butanal	0.06 ± 0.01	0.07 ± 0.01	0.05 ± 0.00	0.134	0.05 ± 0.00	0.06 ± 0.01	0.06 ± 0.01	0.542	0.58 ± 0.32 ^a^	0.33 ± 0.01 ^ab^	0.04 ± 0.00 ^b^	0.034
3-Methyl-3-buten-1-ol	0.05 ± 0.02 ^b^	0.05 ± 0.02 ^ab^	0.08 ± 0.00 ^a^	0.035	0.07 ± 0.00 ^ab^	0.06 ± 0.02 ^b^	0.09 ± 0.01 ^a^	0.053	0.10 ± 0.01 ^b^	0.13 ± 0.00 ^a^	0.1 ± 0.01 ^b^	0.006
3-Methylbutan-1-ol dimer	0.12 ± 0.06	0.15 ± 0.07	0.13 ± 0.01	0.876	0.17 ± 0.02	0.14 ± 0.07	0.12 ± 0.06	0.584	0.19 ± 0.02 ^b^	0.79 ± 0.01 ^a^	0.13 ± 0.02 ^c^	0.000
3-Methylbutan-1-ol monomer	0.66 ± 0.21	0.52 ± 0.15	0.43 ± 0.02	0.251	0.46 ± 0.06	0.39 ± 0.13	0.34 ± 0.11	0.418	0.88 ± 0.20 ^b^	3.52 ± 0.03 ^a^	0.46 ± 0.09 ^c^	0.000
Acetaldehyde	2.40 ± 0.12 ^c^	3.79 ± 0.13 ^b^	5.48 ± 0.36 ^a^	0.000	2.23 ± 0.16 ^b^	3.09 ± 0.28 ^a^	2.77 ± 0.25 ^ab^	0.012	1.62 ± 0.08 ^b^	1.11 ± 0.08 ^b^	3.59 ± 0.63 ^a^	0.000
Acetic acid dimer	1.54 ± 0.48	1.01 ± 0.12	0.83 ± 0.03	0.054	0.71 ± 0.04	0.63 ± 0.16	0.53 ± 0.11	0.222	0.52 ± 0.07 ^a^	0.27 ± 0.07 ^b^	0.48 ± 0.07 ^a^	0.010
Acetic acid monomer	11.39 ± 1.34 ^a^	9.8 ± 0.73 ^a^	7.30 ± 0.35 ^b^	0.004	7.43 ± 0.30	7.54 ± 0.59	6.47 ± 0.92	0.167	6.50 ± 0.52 ^a^	3.88 ± 0.04 ^c^	5.12 ± 0.35 ^b^	0.000
Acetoin dimer	0.35 ± 0.11 ^b^	0.36 ± 0.1 ^b^	3.35 ± 0.29 ^a^	0.000	0.47 ± 0.09	0.37 ± 0.13	0.54 ± 0.21	0.449	1.17 ± 0.42 ^b^	2.53 ± 0.06 ^a^	1.41 ± 0.59 ^b^	0.015
Acetoin monomer	3.19 ± 0.26 ^b^	2.69 ± 0.09 ^c^	13.03 ± 0.60 ^a^	0.000	4.32 ± 1.05 ^ab^	3.46 ± 0.08 ^b^	5.99 ± 1.05 ^a^	0.030	9.27 ± 2.05	9.74 ± 0.09	8.48 ± 1.98	0.657
Acetone	14.55 ± 1.26 ^a^	16.86 ± 1.48 ^a^	11.44 ± 0.33 ^b^	0.003	16.13 ± 0.70	17.07 ± 0.42	15.96 ± 1.78	0.480	26.08 ± 1.78 ^a^	21.28 ± 0.28 ^b^	16.5 ± 1.96 ^c^	0.001
Butanol dimer	0.25 ± 0.03 ^a^	0.23 ± 0.04 ^a^	0.11 ± 0.01 ^b^	0.003	0.18 ± 0.04	0.18 ± 0.03	0.39 ± 0.14	0.037	0.20 ± 0.02	0.17 ± 0.02	0.23 ± 0.06	0.252
Butanol monomer	2.29 ± 0.08 ^a^	2.25 ± 0.01 ^a^	1.19 ± 0.01 ^b^	0.000	1.99 ± 0.21 ^b^	2.17 ± 0.06 ^ab^	2.75 ± 0.42 ^a^	0.032	2.18 ± 0.17 ^a^	1.58 ± 0.03 ^b^	2.00 ± 0.37 ^ab^	0.049
Cyclopentanone	0.07 ± 0.02 ^b^	0.11 ± 0.02 ^b^	0.22 ± 0.02 ^a^	0.000	0.09 ± 0.01	0.09 ± 0.01	0.06 ± 0.02	0.080	0.13 ± 0.02 ^a^	0.07 ± 0.01 ^b^	0.07 ± 0.00 ^b^	0.006
dimethyl sulfide	0.77 ± 0.03 ^b^	1.41 ± 0.01 ^a^	0.54 ± 0.02 ^c^	0.000	1.58 ± 0.22 ^a^	1.61 ± 0.04 ^a^	0.70 ± 0.03 ^b^	0.000	1.75 ± 0.21 ^a^	0.84 ± 0.00 ^b^	0.52 ± 0.01 ^c^	0.000
Ethanol	33.61 ± 1.86 ^a^	26.66 ± 1.16 ^b^	21.25 ± 0.74 ^c^	0.000	32.15 ± 1.26 ^b^	27.73 ± 1.81 ^c^	36.21 ± 0.55 ^a^	0.001	15.30 ± 2.79 ^b^	18.26 ± 0.11 ^b^	30.53 ± 2.64 ^a^	0.000
Ethyl 2-hydroxypropanoate	1.31 ± 0.49 ^a^	0.82 ± 0.09 ^ab^	0.53 ± 0.03 ^b^	0.042	0.51 ± 0.06	0.46 ± 0.11	0.31 ± 0.11	0.095	0.48 ± 0.03 ^a^	0.17 ± 0.06 ^c^	0.34 ± 0.02 ^b^	0.000
Heptanal	0.27 ± 0.05	0.26 ± 0.01	0.22 ± 0.01	0.143	0.28 ± 0.03 ^a^	0.26 ± 0.02 ^ab^	0.21 ± 0.03 ^b^	0.043	0.31 ± 0.02 ^b^	0.16 ± 0.02 ^c^	0.46 ± 0.01 ^a^	0.000
Methyl acetate	0.04 ± 0.01 ^ab^	0.05 ± 0.01 ^a^	0.03 ± 0.01 ^b^	0.029	0.05 ± 0.01 ^a^	0.07 ± 0.01 ^a^	0.03 ± 0.00 ^b^	0.001	0.18 ± 0.03 ^a^	0.13 ± 0.01 ^a^	0.04 ± 0.01 ^b^	0.000
Propanal	3.35 ± 0.81 ^a^	2.96 ± 0.24 ^a^	0.58 ± 0.07 ^b^	0.001	2.28 ± 0.59 ^a^	1.85 ± 0.27 ^ab^	1.07 ± 0.33 ^b^	0.031	2.29 ± 0.58 ^a^	1.24 ± 0.06 ^b^	0.11 ± 0.01 ^c^	0.001

Lowercase letters: the same lowercase letters or no letters indicate no significant difference (*p* > 0.05), and different lowercase letters indicate significant difference (*p* < 0.05).

## Data Availability

Data are contained within the article and Supplementary Materials.

## Supplementary Materials

The following supporting information can be downloaded at: https://www.mdpi.com/article/10.3390/ani14142113/s1, Table S1: Effect of three dietary PKM levels on the composition and content of subcutaneous fat FAs in Tibetan sheep; Table S2: Effects of three dietary PKM levels on FAs composition and content of tail fat in Tibetan sheep; Table S3: Effects of three dietary PKM levels on FAs composition and content of intermuscular fat in Tibetan sheep; Table S4: Differential changes in the composition and content of FAs in different parts of adipose tissue of Tibetan sheep when 0% PKM was added to the diets; Table S5: Differential changes in the composition and content of FAs in different parts of adipose tissue of Tibetan sheep when 15% PKM was added to the diets; Table S6: Differential changes in the composition and content of FAs in different parts of adipose tissue of Tibetan sheep when 18% PKM was added to the diets.
