# Supplementary material for: Fatty Acids and Volatile Flavor Components of Adipose Tissue from Local Tibetan Sheep in Qinghai with Dietary Supplementation of Palm Kernel Meal (PKM)
Source: Animals (Basel). 2024 Jul 20;14(14):2113. doi: 10.3390/ani14142113 (PMC11274258; doi:10.3390/ani14142113)
Supplement: Supplementary file 1 [file animals-14-02113-s001.zip › animals-3070461-supplementary.pdf]

Table S1. Effect of three dietary PKM levels on the composition and content of subcutaneous fat FAs in Tibetan sheep

Item	Diet[1]			SEM	P-value
FAs	P0	P15	P18		
C6:0	0.03±0.01	0.03±0.00	0.04±0.02	0.01	0.454
C8:0	0.09±0.0.05	0.06±0.01	0.07±0.02	0.03	0.612
C10:0	11.67±4.86	14.34±5.05	11.66±7.46	4.82	0.752
C11:0	0.42±0.07	0.59±0.33	0.36±0.22	0.19	0.426
C12:0	12.12±1.96[b]	57.55±37.45[a]	40.32±18.42[a]	19.70	0.017
C13:0	2.72±0.46	3.33±1.12	2.28±1.10	0.77	0.389
C14:0	483.64±27.73[a]	963.26±240.15[b]	949.53±247.13[b]	162.97	0.016
C15:0	125.11±19.67	146.26±35.38	113.16±37.67	26.06	0.444
C16:0	3115.88±261.24[b]	3988.93±392.32[ab]	4377.98±727.93[a]	408.80	0.042
C17:0	428.31±34.36	334.82±11.48	352.22±71.00	37.58	0.108
C18:0	3225.28±464.38	2772.43±1024.23	3268.19±299.48	548.61	0.557
C20:0	30.24±7.60	27.62±7.67	19.97±3.31	5.32	0.191
C21:0	0.09±0.02	0.13±0.02	0.13±0.04	0.02	0.179
C22:0	2.06±0.49	2.12±0.70	1.96±1.42	0.41	0.971
C23:0	0.75±0.12	0.61±0.07	0.60±0.09	0.08	0.195
C24:0	1.02±0.10	0.87±0.05	0.84±0.08	0.07	0.081
C14:1N5	13.13±5.98	72.27±67.15	41.64±18.39	32.94	0.068
C15:1N5	15.25±3.74	18.40±2.97	19.56±3.00	2.66	0.315
C16:1N7	319.86±72.87	674.08±346.96	569.12±202.42	192.45	0.138
C17:1N7	200.67±548.25	227.10±96.86	190.11±34.00	54.86	0.860
C18:1N9	5546.71±779.17	6732.50±437.32	7436.35±1634.47	878.11	0.136
C20:1N9	16.77±5.67[a]	5.86±1.61[b]	19.58±4.69[a]	3.55	0.004
C22:1N9	25.07±9.79	19.21±7.99	22.10±1.32	5.99	0.590
C18:2N6	299.44±55.64	355.76±26.78	369.69±116.17	62.02	0.506
C20:2N6	13.83±1.02	12.79±0.69	14.04±3.34	1.68	0.768
C22:2N6	0.94±0.42	0.70±0.09	0.61±0.22	0.23	0.353
C18:3N3	33.78±8.98	53.67±10.27	46.02±16.46	10.08	0.187
C18:3N6	1.36±0.35	1.81±0.34	1.57±0.25	0.26	0.269
C20:3N6	0.49±0.12	0.53±0.04	0.56±0.16	0.10	0.763
C20:3N3	0.59±0.25	0.71±0.20	0.76±0.17	0.17	0.539
C20:4N6	17.97±5.22	18.10±3.67	18.84±7.03	4.48	0.993
C20:5N3	0.18±0.06	0.26±0.04	0.22±0.04	0.04	0.246
C22:5N3	2.20±0.95	2.30±0.81	1.81±0.73	0.68	0.774
C22:6N3	4.02±0.50	6.85±2.28	5.86±1.71	1.37	0.131
C18:2TTN6	26.35±7.24[b]	42.54±5.00[a]	34.81±4.23[ab]	4.60	0.040

Table S2. Effects of three dietary PKM levels on FAs composition and content of tail fat in Tibetan sheep

Item	Diet			SEM	P-value
FAs	W0	W15	W18		

C6:0	0.03±0.01	0.03±0.01	0.04±0.00	0.00	0.079
C8:0	0.06±0.02	0.04±0.00	0.07±0.02	0.01	0.099
C10:0	11.79±4.45	7.85±1.75	10.10±2.88	2.63	0.352
C11:0	0.46±0.20	0.35±0.05	0.38±0.03	0.10	0.659
C12:0	12.04±3.50^{b}	29.26±6.19^{a}	42.74±7.84^{a}	4.99	0.001
C13:0	2.40±1.13	2.33±0.43	2.51±0.21	0.58	0.882
C14:0	473.36±75.47^{b}	667.65±108.92^{ab}	865.89±69.64^{a}	70.57	0.005
C15:0	119.74±43.05	111.76±22.91	122.38±18.26	24.55	0.886
C16:0	3275.53±358.91	3309.86±649.47	3993.67±489.82	419.14	0.235
C17:0	431.93±87.72	338.42±41.06	360.76±56.20	52.79	0.257
C18:0	2759.10±428.78	2824.02±315.44	2784.23±378.84	308.00	0.971
C20:0	25.33±5.73	25.75±5.84	19.78±3.47	4.19	0.363
C21:0	0.07±0.02^{b}	0.09±0.02^{ab}	0.13±0.01^{a}	0.02	0.037
C22:0	1.83±0.19	1.52±0.32	2.22±0.45	0.28	0.111
C23:0	0.60±0.07	0.54±0.16	0.61±0.08	0.09	0.663
C24:0	0.93±0.07^{a}	0.75±0.09^{b}	0.86±0.03^{ab}	0.06	0.047
C14:1N5	25.53±15.35	31.33±17.85	57.57±14.19	12.96	0.129
C15:1N5	17.09±2.05^{ab}	15.09±2.83^{b}	21.79±1.95^{a}	1.88	0.041
C16:1N7	432.29±134.31	407.49±185.94	667.67±141.64	127.08	0.177
C17:1N7	287.25±121.50	175.66±73.28	283.66±103.17	82.70	0.316
C18:1N9	7058.02±987.19	6086.40±1804.64	8803.51±1822.81	1295.62	0.189
C20:1N9	11.70±3.47	7.82±3.25	16.99±2.02	2.44	0.058
C22:1N9	12.43±0.64	17.16±3.83	15.90±4.31	2.74	0.255
C18:2N6	274.78±51.76	313.36±45.74	363.28±37.58	37.07	0.143
C20:2N6	13.37±1.45	10.73±3.06	15.59±1.60	1.76	0.094
C22:2N6	0.45±0.12	0.52±0.16	0.45±0.22	0.14	0.797
C18:3N3	32.73±7.82	41.35±6.62	50.46±5.24	5.42	0.054
C18:3N6	1.58±0.26	1.42±0.38	2.13±0.17	0.23	0.068
C20:3N3	0.58±0.17	0.52±0.03	0.93±0.26	0.15	0.057
C20:3N6	0.38±0.12	0.44±0.16	0.57±0.08	0.10	0.222
C20:4N6	1.47±0.39	1.49±0.31	1.81±0.06	2.39	0.347
C20:5N3	0.20±0.03	0.18±0.06	0.22±0.06	0.04	0.661
C22:5N3	1.86±0.61	2.19±0.06	2.28±0.37	0.34	0.424
C22:6N3	3.97±0.19	5.04±1.30	5.36±1.65	0.99	0.372
C18:2TTN6	30.52±8.51	37.31±14.75	49.62±6.02	8.51	0.150

Table S3. Effects of three dietary PKM levels on FAs composition and content of intermuscular fat in Tibetan sheep

Item	Diet			SEM	P-value
FAs	J0	J15	J18		
C6:0	0.07±0.03	0.06±0.04	0.04±0.01	0.02	0.381
C8:0	0.05±0.01	0.07±0.02	0.09±0.05	0.03	0.392
C10:0	3.79±6.43	8.51±7.67	13.73±11.10	7.05	0.293

C11:0	0.18±0.27	0.24±0.20	0.39±0.32	0.22	0.547
C12:0	5.81±6.89	28.35±19.35	56.83±42.44	22.23	0.084
C13:0	1.14±1.58	1.63±1.24	2.69±2.07	1.36	0.468
C14:0	307.26±210.06	679.91±350.90	1088.92±572.75	331.76	0.113
C15:0	65.73±67.31	77.02±44.62	105.45±57.53	46.74	0.578
C16:0	2428.44±1070.06	3352.09±1180.16	4175.87±1026.08	893.26	0.213
C17:0	218.00±225.57	207.64±111.19	254.38±82.42	124.76	0.737
C18:0	1775.52±1202.69	2695.76±1221.40	2920.60±807.66	893.26	0.380
C20:0	11.15±11.92	21.07±12.52	17.34±7.64	8.91	0.447
C21:0	0.07±0.03	0.09±0.02	0.11±0.06	0.03	0.670
C22:0	1.08±0.10	1.21±0.42	1.48±0.69	0.59	0.670
C23:0	0.30±0.27	0.48±0.24	0.54±0.28	0.21	0.456
C24:0	0.52±0.34	0.73±0.27	0.65±0.27	0.24	0.625
C14:1N5	7.33±5.48	21.39±14.29	31.22±14.06	9.80	0.066
C15:1N5	6.37±2.46	12.84±5.60	19.67±9.76	5.43	0.113
C16:1N7	224.54±104.07	337.42±164.83	435.65±137.95	112.57	0.222
C17:1N7	110.93±87.36	98.22±45.59	127.47±35.37	49.35	0.731
C18:1N9	3853.97±1480.61	4970.72±1536.11	6199.56±1381.04	1198.06	0.229
C20:1N9	7.48±8.20	5.33±5.37	18.25±12.98	7.67	0.387
C22:1N9	12.48±15.87	17.34±12.56	27.85±19.52	13.25	0.507
C18:2N6	176.53±107.96	264.82±112.98	385.26±232.96	132.24	0.380
C20:2N6	8.95±4.28	8.92±2.97	12.07±3.18	2.88	0.486
C22:2N6	0.78±0.41	0.73±0.24	0.55±0.02	0.22	0.587
C18:3N3	14.10±11.78	33.52±17.97	47.85±32.94	18.54	0.249
C18:3N6	0.75±0.40	1.10±0.42	1.52±0.70	0.43	0.318
C20:3N6	0.35±0.12	0.37±0.12	0.38±0.07	0.19	0.288
C20:3N3	0.28±0.10	0.39±0.18	0.64±0.36	0.08	0.871
C20:4N6	20.96±1.63	17.46±2.17	17.25±3.42	2.06	0.222
C20:5N3	0.10±0.05	0.14±0.06	0.13±0.03	0.04	0.220
C22:5N3	1.44±0.67	1.89±0.64	1.22±0.53	0.50	0.461
C22:6N3	2.01±1.74	4.42±2.85	5.19±3.19	2.18	0.481
C18:2TTN6	12.55±8.96	24.15±11.93	26.52±8.70b	8.14	0.262

Table S4. Differential changes in the composition and content of FAs in different parts of adipose tissue of Tibetan sheep when 0% PKM was added to the diets

Item	Adipose tissue			SEM	P-value
FAs	P0	W0	J0		
C6:0	0.03±0.01	0.03±0.01	0.07±0.03	0.01	0.050
C8:0	0.09±0.0.05	0.06±0.02	0.05±0.01	0.03	0.440
C10:0	11.67±4.86	11.79±4.45	3.79±6.43	4.34	0.140
C11:0	0.42±0.07	0.46±0.20	0.18±0.27	0.16	0.124
C12:0	12.12±1.96	12.04±3.50	5.81±6.89	3.76	0.139
C13:0	2.72±0.46	2.40±1.13	1.14±1.58	0.94	0.141
C14:0	483.64±27.73	473.36±75.47	307.26±210.06	106.03	0.172
C15:0	125.11±19.67	119.74±43.05	65.73±67.31	38.79	0.169
C16:0	3115.88±261.24	3275.53±358.91	2428.44±1070.06	546.12	0.254

C17:0	428.31±34.36	431.93±87.72	218.00±225.57	115.24	0.122
C18:0	3225.28±464.38	2759.10±428.78	1775.52±1202.69	640.48	0.121
C20:0	30.24±7.60	25.33±5.73	11.15±11.92	7.19	0.074
C21:0	0.09±0.02	0.07±0.02	0.07±0.03	0.02	0.639
C22:0	2.06±0.49	1.83±0.19	1.08±0.10	0.14	0.153
C23:0	0.75±0.12	0.60±0.07	0.30±0.27	0.17	0.067
C24:0	1.02±0.10	0.93±0.07	0.52±0.34	0.52	0.062
C14:1N5	13.13±5.98	25.53±15.35	7.33±5.48	8.18	0.090
C15:1N5	15.25±3.74^{a}	17.09±2.05^{a}	6.37±2.4^{b}	2.32	0.006
C16:1N7	319.86±72.87	432.29±134.31	224.54±104.07	87.15	0.117
C17:1N7	200.67±548.25	287.25±121.50	110.93±87.36	75.13	0.098
C18:1N9	5546.71±779.17^{ab}	7058.02±987.19^{a}	3853.97±1480.61^{b}	915.77	0.042
C20:1N9	16.77±5.67	11.70±3.47	7.48±8.20	4.97	0.162
C22:1N9	25.07±9.79	12.43±0.64	12.48±15.87	8.79	0.229
C18:2N6	299.44±55.64	274.78±51.76	176.53±107.96	62.24	0.143
C20:2N6	13.83±1.02	13.37±1.45	8.95±4.28	2.18	0.107
C22:2N6	0.94±0.42	0.45±0.12	0.78±0.41	0.28	0.169
C18:3N3	33.78±8.98	32.73±7.82	14.10±11.78	7.90	0.065
C18:3N6	1.36±0.35^{a}	1.58±0.26^{a}	0.75±0.40^{b}	0.28	0.049
C20:3N3	0.49±0.12	0.58±0.17	0.35±0.12	0.15	0.060
C20:3N6	0.59±0.25	0.38±0.12	0.28±0.10	0.10	0.378
C20:4N6	17.97±5.22	1.47±0.39	20.96±1.63	3.17	0.243
C20:5N3	0.18±0.06	0.20±0.03	0.10±0.05	0.04	0.063
C22:5N3	2.20±0.95	1.86±0.61	1.44±0.67	0.62	0.545
C22:6N3	4.02±0.50	3.97±0.19	2.01±1.74	0.86	0.101
C18:2TTN6	26.35±7.24	30.52±8.51	12.55±8.96	6.75	0.057

Table S5. Differential changes in the composition and content of FAs in different parts of adipose tissue of Tibetan sheep when 15% PKM was added to the diets

Item	Adipose tissue			SEM	P-value
FAs	P15	W15	J15		
C6:0	0.03±0.00	0.03±0.01b	0.06±0.04	0.02	0.168
C8:0	0.06±0.01	0.04±0.00	0.07±0.02	0.01	0.078
C10:0	14.34±5.05	7.85±1.75	8.51±7.67	4.41	0.463
C11:0	0.59±0.33	0.35±0.05	0.24±0.20	0.18	0.260
C12:0	57.55±37.45	29.26±6.19	28.35±19.35	20.09	0.385
C13:0	3.33±1.12	2.33±0.43	1.63±1.24	0.81	0.288
C14:0	963.26±240.15	667.65±108.92	679.91±350.90	206.92	0.417
C15:0	146.26±35.38	111.76±22.91	77.02±44.62	28.93	0.197
C16:0	3988.93±392.32	3309.86±649.47	3352.09±1180.16	661.40	0.545
C17:0	334.82±11.48	338.42±41.06	207.64±111.19	56.14	0.187
C18:0	2772.43±1024.23	2824.02±315.44	2695.76±1221.40	766.00	0.923
C20:0	27.62±7.67	25.75±5.84	21.07±12.52	7.45	0.594
C21:0	0.13±0.02	0.09±0.02	0.09±0.02	0.02	0.137
C22:0	2.12±0.70	1.52±0.32	1.21±0.42	0.42	0.166
C23:0	0.61±0.07	0.54±0.16	0.48±0.24	0.14	0.567
C24:0	0.87±0.05	0.75±0.09	0.73±0.27	0.14	0.506
C14:1N5	72.27±67.15	31.33±17.85	21.39±14.29	33.44	0.248
C15:1N5	18.40±2.97	15.09±2.83	12.84±5.60	3.27	0.354
C16:1N7	674.08±346.96	407.49±185.94	337.42±164.83	20.18	0.256
C17:1N7	227.10±96.86	175.66±73.28	98.22±45.59	61.16	0.122
C18:1N9	6732.50±437.32	6086.40±1804.64	4970.72±1536.11	1136.04	0.329
C20:1N9	5.86±1.61	7.82±3.25	5.33±5.37	3.06	0.492
C22:1N9	19.21±7.99	17.16±3.83	17.34±12.56	7.25	0.807
C18:2N6	355.76±26.78	313.36±45.74	264.82±112.98	58.83	0.398
C20:2N6	12.79±0.69	10.73±3.06	8.92±2.97	2.03	0.248

C22:2N6	0.70±0.09	0.52±0.16	0.73±0.24	0.14	0.288
C18:3N3	53.67±10.27	41.35±6.62	33.52±17.97	10.24	0.310
C18:3N6	1.81±0.34	1.42±0.38	1.10±0.42	0.31	0.189
C20:3N3	0.53±0.04	0.52±0.03	0.37±0.12	0.13	0.164
C20:3N6	0.71±0.20	0.44±0.16	0.39±0.18	0.09	0.305
C20:4N6	18.10±3.67	1.49±0.31	17.46±2.17	2.49	0.397
C20:5N3	0.26±0.04	0.18±0.06	0.14±0.06	0.04	0.116
C22:5N3	2.30±0.81	2.19±0.06	1.89±0.64	0.49	0.669
C22:6N3	6.85±2.28	5.04±1.30	4.42±2.85	1.83	0.409
C18:2TTN6	42.54±5.00	37.31±14.75	24.15±11.93	9.25	0.212

Table S6. Differential changes in the composition and content of FAs in different parts of adipose tissue of Tibetan sheep when 18% PKM was added to the diets

Item	Adipose tissue			SEM	P-value
FAs	P18	W18	J18		
C6:0	0.04±0.02	0.04±0.00	0.04±0.01	0.01	0.688
C8:0	0.07±0.02	0.07±0.02	0.09±0.05	0.03	0.800
C10:0	11.66±7.46	10.10±2.88	13.73±11.10	6.45	0.932
C11:0	0.36±0.22	0.38±0.03	0.39±0.32	0.18	0.848
C12:0	40.32±18.42	42.74±7.84	56.83±42.44	22.12	0.979
C13:0	2.28±1.10	2.51±0.21	2.69±2.07	1.11	0.916
C14:0	949.53±247.13	865.89±69.64	1088.92±572.75	295.88	0.941
C15:0	113.16±37.67	122.38±18.26	105.45±57.53	33.54	0.746
C16:0	4377.98±727.93	3993.67±489.82	4175.87±1026.08	636.42	0.851
C17:0	352.22±71.00	360.76±56.20	254.38±82.42	57.72	0.181
C18:0	3268.19±299.48	2784.23±378.84	2920.60±807.66	443.60	0.587
C20:0	19.97±3.31	19.78±3.47	17.34±7.64	4.25	0.685
C21:0	0.13±0.04	0.13±0.01	0.11±0.06	0.03	0.573
C22:0	1.96±1.42	2.22±0.45	1.48±0.69	0.39	0.287
C23:0	0.60±0.09	0.61±0.08	0.54±0.28	0.14	0.730
C24:0	0.84±0.08	0.86±0.03	0.65±0.27	0.13	0.318
C14:1N5	41.64±18.39	57.57±14.19	31.22±14.06	12.80	0.259
C15:1N5	19.56±3.00	21.79±1.95	19.67±9.76	4.90	0.794
C16:1N7	569.12±202.42	667.67±141.64	435.65±137.95	133.39	0.284
C17:1N7	190.11±34.00	283.66±103.17	127.47±35.37	53.85	0.058
C18:1N9	7436.35±1634.47	8803.51±1822.81	6199.56±1381.04	1325.09	0.232
C20:1N9	19.58±4.69	16.99±2.02	18.25±12.98	6.58	0.814
C22:1N9	22.10±1.32	15.90±4.31	27.85±19.52	9.44	0.707
C18:2N6	369.69±116.17	363.28±37.58	385.26±232.96	2.30	0.966
C20:2N6	14.04±3.34	15.59±1.60	12.07±3.18	123.99	0.368
C22:2N6	0.61±0.22	0.45±0.22	0.55±0.02	0.15	0.494
C18:3N3	46.02±16.46	50.46±5.24	47.85±32.94	17.53	0.834
C18:3N6	1.57±0.25	2.13±0.17	1.52±0.70	0.36	0.357
C20:3N3	0.56±0.16	0.93±0.26	0.38±0.07	0.23	0.446
C20:3N6	0.76±0.17	0.57±0.08	0.64±0.36	0.09	0.108
C20:4N6	18.84±7.03	1.81±0.06	17.25±3.42	3.70	0.957
C20:5N3	0.22±0.04	0.22±0.06	0.13±0.03	0.03	0.054
C22:5N3	1.81±0.73	2.28±0.37	1.22±0.53	0.46	0.178
C22:6N3	5.86±1.71	5.36±1.65	5.19±3.19	1.87	0.819
C18:2TTN6	34.81±4.23^{ab}	49.62±6.02^{a}	26.52±8.70^{b}	5.37	0.038
